# Systematic review and meta-analysis of standalone digital interventions for cognitive symptoms in people without dementia

**DOI:** 10.1038/s41746-024-01280-9

**Published:** 2024-10-10

**Authors:** Veronica Cabreira, Tim Wilkinson, Lisbeth Frostholm, Jon Stone, Alan Carson

**Affiliations:** 1 Centre for Clinical Brain Sciences, University of Edinburgh, Edinburgh, UK; 2 Department of Clinical Medicine, Aarhus University, Aarhus, Denmark; 3 Department of Functional Disorders and Psychosomatics, Aarhus University Hospital, Aarhus, Denmark

**Keywords:** Neurological disorders, Neurological manifestations, Therapeutics

## Abstract

Cognitive symptoms are prevalent across neuropsychiatric disorders, increase distress and impair quality of life. Self-guided digital interventions offer accessibility, scalability, and may overcome the research-to-practice treatment gap. Seventy-six trials with 5214 participants were identified. A random-effects meta-analysis investigated the effects of all digital self-guided interventions, compared to controls, at post-treatment. We found a small-to-moderate positive pooled effect on cognition (*k* = 71; *g* = −0.51, 95%CI −0.64 to −0.37; *p* < 0.00001) and mental health (*k* = 30; *g* = −0.41, 95%CI −0.60 to −0.22; *p* < 0.0001). Positive treatment effects on fatigue (*k* = 8; *g* = −0.27, 95%CI −0.53 to −0.02; *p* = 0.03) and quality of life (*k* = 22; *g* = −0.17, 95%CI −0.34 to −0.00; *p* = 0.04) were only marginally significant. No significant benefit was found for performance on activities of daily living. Results were independent of control groups, treatment duration, risk of bias and delivery format. Self-guided digital transdiagnostic interventions may benefit at least a subset of patients in the short run, yet their impact on non-cognitive outcomes remains uncertain.

## Introduction

Cognitive symptoms are common across several neuropsychiatric disorders, with only a minority stemming from progressive underlying neurodegenerative diseases^1^. Today, only 24–53% of the attendees of memory services targeting individuals under 65 have a neurodegenerative disease^2,3^. Adult patients experiencing cognitive symptoms but without functional impairment are often arbitrarily categorized as having subjective cognitive decline (SCD) or mild cognitive impairment (MCI)^4–6^. Although these terms are aetiologically agnostic, they can mislead patients into believing they will inevitably worsen and doctors into expecting their patients to develop Alzheimer’s disease^1,7,8^. In fact, most of these cognitive symptoms result from various reversible causes like sleep disturbances or medication side-effects and are also common in other conditions such as post-mild traumatic brain injury^9^, functional disorders^10^, multiple sclerosis, stroke^11^ and Parkinson’s disease^1,3^. Subjective cognitive symptoms can also develop in adult healthy individuals with heightened awareness of their memory symptoms, especially with aging^12–14^. Up to 30% of the population will experience cognitive symptoms at some point in life^14^.

Regardless of the aetiology, cognitive symptoms are linked with reduced self-esteem and quality of life^15,16^, mood and anxiety disorders^15^, reduced productivity^15^ and substantial healthcare costs^3,17^. In many instances, anticipatory anxiety, fear of failing, concerns related to dementia, and the need for repeated medical investigations further exacerbate attention dysregulation and thinking errors, fuelling the symptoms^3^. Although prognostic studies are scarce for patients without dementia, research suggests that cognitive symptoms are unlikely to improve over time or with simple reassurance^18,19^, and are negatively associated with employment outcomes^18^. Despite this, memory symptoms remain largely undertreated^20,21^. Addressing cognitive symptoms effectively is a public health priority^17^.

Effective treatment is hindered by barriers like financial costs, time constraints, and limited specialized services^22,23^. Moreover, the common misconception that cognitive symptoms form a continuum from subjective complaints to Alzheimer’s disease persists, despite the fact that many patients do not conform to this trajectory^5,12^ This has directed the focus of therapeutic research towards Alzheimer’s disease, for which symptomatic and disease-modifying drugs are now available^23^, leaving many patients with cognitive symptoms unsupported. Hence, current practice favours discharging a significant proportion of individuals with cognitive complaints after the exclusion of a dementia diagnosis^4^. Increasing access to accessible and evidence-based treatments may be a promising avenue to overcome the research-to-practice gap in this population.

Remote self-help and internet-based interventions offer scalability and flexibility to adjust to patients’ tolerance and schedules while overcoming some of the reported treatment barriers^24–27^. They often do not require extra costs or equipment as many technologies are an integral part of everyday life. Health institutions have long utilized computerized interventions like cognitive training and rehabilitation programmes^9,28–30^ for neurological and mental health conditions^31–33^. Further, the growth in smartphones and virtual reality technologies opens a window for innovative treatment options^24,25^. However, potential challenges and drawbacks include technical difficulties, technology requirements and digital literacy levels limiting the effective use of the materials provided, limited tailoring, data-security concerns, and reduced engagement or high attrition rates, which are at least partially driven by reduced therapist contact, perceived lack of support and reduced internal locus of control^34–36^. Also, despite the growing market for digital interventions and smartphone apps targeting cognitive symptoms, the effectiveness and safety of standalone digital therapeutic options for cognitive symptoms are still unclear. Interestingly, data shows that adoption, implementation and patient recommendation by healthcare professionals are highly dependent on credibility and demonstrated clinical effectiveness^37^.

The evidence to date on self-help remote interventions for cognitive symptoms is summarized in various meta-analyses for various disorders including SCD^21^, MCI^38,39^, post-stroke cognitive impairment^40–42^, post-traumatic brain injury^9,43^, cancer patients^44^, healthy older adults^45^, ADHD^46^, and other neurological conditions^30,47^, with mixed efficacy. The within-group pre-post and between-group effect sizes vary widely between the studies, with most studies showing small-to moderate effects on memory, attention, processing speed, and executive functions. Notably, not all the studies included are randomized controlled trials (RCTs), limiting analysis of between-group differences. The interventions included are heterogeneous (combination of cognitive interventions plus exercise^48^, guided and non-guided), which restricts the data on potentially scalable standalone self-help interventions. Moreover, these meta-analyses are often limited to specific frameworks (e.g. meta-analysis only in computerized cognitive rehabilitation or virtual reality^49^) and populations (e.g. MCI or Parkinson’s disease only), hindering comparison between therapeutic approaches to support clinical decisions, and failing to acknowledge that transdiagnostic mechanisms account for cognitive symptoms across different populations. No meta-analyses to date have analyzed the between-group effects across different technologies and therapeutic approaches for a range of transdiagnostic non-neurodegenerative cognitive symptoms. Additionally, there is inconsistent and generally poor evidence for transfer effects of cognitive digital interventions to clinically meaningful outcomes like activities of daily living, psychological wellbeing and quality of life^50^. Characterizing the efficacy of standalone self-guided digital interventions for cognitive symptoms is important, given the potential for increased relevance of these tools in clinical practice^1^.

The aim of this systematic review and meta-analysis is to investigate the effectiveness of standalone self-guided digital interventions in improving cognitive, physical, activities of daily living, mental health and quality of life outcomes, among patients with transdiagnostic cognitive symptoms without dementia, in comparison to control conditions. Secondarily, we aim to explore whether factors like population studied, trial design features (e.g. treatment duration and control groups), therapeutic frameworks (e.g. cognitive training, cognitive rehabilitation, videogames, cognitive behavioural therapy (CBT)), and delivery methods (e.g. app, computer, virtual reality and game consoles) influence any cognitive benefits observed.

## Results

### Study selection

**Fig. 1:**
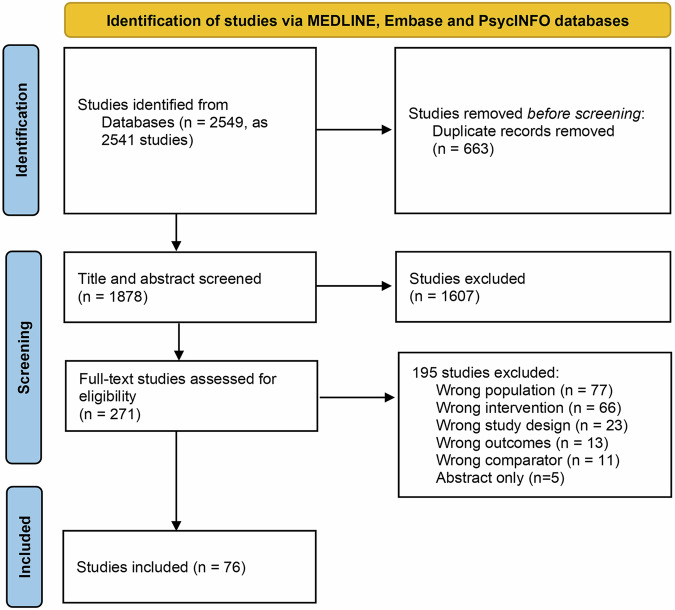
PRISMA flow diagram. The flow diagram shows the number of records identified, included and excluded at the different stages of the systematic review. A total of 2541 studies were retrieved from the electronic databases. After removal of duplicates and exclusion based on title and abstract screening, 271 full-text studies were assessed for eligibility. Finally, 76 trials fulfilling all inclusion criteria were included.

A total of 2541 studies were retrieved from the electronic databases. After removal of duplicates and exclusion based on title and abstract screening, 271 full-text studies were assessed for eligibility. Finally, 76 trials fulfilling all inclusion criteria were included. Inter-rater reliability for of title and abstract screening and full-text eligibility showed a Cohen’s kappa of 0.63 and 0.92, respectively, indicating good to excellent agreement. The study selection process and reasons for exclusion are displayed in Fig. 1.

### Characteristics of included studies

The 76 RCTs included a total of 5214 participants. Mean age of participants was 58 years (SD = 15). Sample sizes ranged from 20 to 243 patients (median = 55) and date of publication from 2007 to 2024. The RCTs were conducted in Europe (*k* = 33, Italy (*k* = 9), Greece (*k* = 6), Netherlands (*k* = 4), UK (*k* = 4), Germany (*k* = 2), Sweden (*k* = 3), and one from Belgium, Czech Republic, Finland, Spain and Slovakia each), the United States (*k* = 13), South Korea (*k* = 9), China (*k* = 6), Australia (*k* = 2), Canada (*k* = 3), Turkey (*k* = 3), Taiwan (*k* = 3), Iran (*k* = 1), Israel (*k* = 1), and Colombia (*k* = 1). One trial was multicentric^51^. The 76 included studies used different types of comparators: *k* = 32 active control groups (*k* = 12 computer activities like watching news, online searching, or online crosswords/sham games; *k* = 3 educational content with information about the brain and general health; *k* = 14 face-to-face standard treatment with a therapist or group therapy; *k* = 3 'paper and pencil activities'), *k* = 26 treatment as usual/standard care, and *k* = 19 waitlist controls. Supplementary Table 1 provides an overview of the individual study characteristics.

Intervention frameworks consisted of cognitive training (*k* = 33), cognitive rehabilitation (*k* = 25), virtual reality (*k* = 12), videogames (partially based on cognitive training) (*k* = 4), internet-delivered courses including principles of Cognitive Behavioural Therapy (CBT) and support for routine structuring and organization skills (*k* = 2) and cognitive remediation (*n* = 1). In a further exploration of the studies, we reported the digital intervention frameworks, mode of delivery and software details for all the included RCTs (see Table 1).

**Table 1:** Main cognitive interventions tested across all trials, with specific examples

Intervention	Treatment process	Delivery mode	Examples of software
Cognitive training^52,54–61,63–66,71,79,80,91,95,97,99,101,103,107,110,111,113–115,121,124,125,157^	Repeated practice on standardised cognitive exercises targeting specific cognitive domains. Purpose: stimulating cognitive function by targeting neural networks and improving plasticity (often adjusted difficulty level).	Compact disk (*n* = 2), computerized (*n* = 28), app (*n* = 1)	Cogmed QM, SOCIABLE, Posit Science Corporation, CoRe, Cogmed, Brainstim, GRADIOR, Lumosity, FESKITS Estimulación Cognitiva, CogniPlus, Spaced Retrieval-based Memory Advancement and Rehabilitation Training (USMART), NEUROvitalis, INSIGHT (Posit), Braingymmer, Smartphone-based brain Anti-aging and memory Reinforcement Training (SMART), BrainHQ, VILAT-G, http://app-cerebroactivo.rhcloud.com, BEYNEX, Neuro-World
Cognitive rehabilitation^32,51,57,78,81,83,85–90,93,98,100,102,105,106,109,112,118–120,122,123^	Activities that are based on assessment and understanding of the patient’s brain-behavioural deficits, complemented by teaching on internal and external memory aids. Purpose: development of behavioural and cognitive strategies that will help retrieval and recall of information, as an attempt to restore memory function using new strategies (‘restitution’ or ‘compensation’).	Computerized (*n* = 23), app (*n* = 1)	NOROSOFT Mental Exercise Program, COGPACK neurorehabilitation programme (Marker Software), Insight neurocognitive program, HAPPY neuron Brain Jogging, RehaCom, Dr. Kawashima’s Brain Training, ERICA platform, MS-Line!, Posit Science’s BrainFitness, CoRe software, ReMind-app, Cognitive vitality training, Lumosity, eReCog, BrainHQ, http://dvbic.dcoe.mil/research/studymanual, 'Niet-Rennen- Maar-Plannen'
Virtual reality^53,68–70,72–77,92,96,104^	Interventions involving a head-mounted display worn by users, which allows them to experience 3D content (eg, videos and games) in an immersive virtual environment. Purpose: promote the ability to switch between different tasks that requires visual ability and attention. Real-time feedback stimulation simulating real-world.	Computerized augmented reality (*n* = 3), Virtual reality system (*n* = 9), mixed (*n* = 1)	Virtools platform, VIVE and Kinect systems, SY Innotech Inc (South Korea), VRRS-Evo, HTC Vive, Cognitive training (COGNIPLAT) Game Platform through augmented reality (https://cogniplat.aegean.gr/Home_Activities/), VRADA
Videogames (including cognitive training)^62,82,94,108^	Different activities and content often delivered in the form of a videogame that intend to improve cognition, but resemble a leisure activity and carry inherent playfulness.	Online games (*n* = 3), Nintendo console (*n* = 1), tablet-based (*n* = 1)	Aquasnap (Cambridge, MyCQ™), 'My Better Mind', Nintendo, Game Show, BEYNEX, EVO Monitor
Internet-delivered courses including CBT^116,117^	A form of psychotherapy attempting to change symptoms by targeting thoughts and behaviours; can be complemented by other technics including organizational skills.	App (*n* = 1), computerized (*n* = 1)	Living SMART, In Focus

Intervention duration ranged from 2 weeks to 6 months (median: 8 weeks). Median number of sessions per week was 3, with a median of 45 minutes per session. The mode of delivery varied: computerized (*n* = 53); virtual reality software/environment (*n* = 13); App/webapp (*n* = 4); videogames or similar (*n* = 4); compact disc (*n* = 2).

As per the inclusion criteria, all studies were based on self-guided interventions (only technical guidance and support allowed) and all studies included cognition as part of their primary outcome. The study outcomes of the 76 included studies were grouped into cognition (*k* = 76), physical function/fatigue (*k* = 12), activities of daily living (*k* = 25), mental health (depression and anxiety) (*k* = 42) and quality of life (*k* = 25) to reflect the variety of outcomes measured (Supplementary tables 2 and 3). All studies reported symptom severity based on rating scales at the end of treatment. Fifty-seven studies reported adherence data as percentage of patients who dropped the study (median dropout rate 13%, range 0–43). Most of the studies did not report data on treatment‐related adverse events so this was not systematically analyzed.

Studies included populations of older patients with cognitive complaints, subjective cognitive decline or MCI (*k* = 28)^52–79^, followed by studies in which cognitive symptoms were related to a history of inflammatory conditions including multiple sclerosis and systemic lupus erythematosus (mean of 13 years since the diagnosis) (*k* = 19)^32,51,80–96^, stroke (from 3 months to 5 years after stroke) (*k* = 8)^97–104^, cancer (from three months to 7 years since diagnosis) (*k* = 8)^105–112^, ADHD (*k* = 5)^113–117^, Parkinson’s disease (mean of 7 years since diagnosis) (*k* = 4)^118–121^, traumatic brain injury (from 25 days to 7 years post-injury) (*k* = 3)^122–124^, and lung transplant (*k* = 1)^125^.

Sixty-nine studies provided enough data and were included in the meta-analyses (Supplementary table 2). For crossover trials (*k* = 3), we only included data from the first period to avoid carry-over effects of the intervention^56,61,108^. For the factorial design trial (*k* = 1), we considered only the effect of the self-guided intervention versus control^55^. For multiple active intervention armed studies (*k* = 15)^51,57,63,64,75,95,99–101,111,112,114,115,117,118^, only the self-guided intervention arm fitting our inclusion criteria was considered against control, except for one trial with two eligible active arms^57^ which were compared against the same control condition (dividing the sample size) to calculate treatment effect size for each intervention^126,127^. This resulted in forty-one head-to-head comparisons with a waitlist/treatment as usual control group, and thirty against active control groups for the outcome cognition; four head-to-head comparisons with a waitlist/treatment as usual control group, and four against an active control group for the outcome fatigue; eight head-to-head comparisons with a waitlist/treatment as usual control group, and thirteen against active control groups for the outcome activities of daily living; seventeen head-to-head comparisons with a waitlist/treatment as usual control group, and thirteen against active control groups for the outcome mental health; thirteen head-to-head comparisons with a waitlist/treatment as usual control group, and nine against active control groups for the outcome quality of life; and twenty-seven head-to-head comparisons with a waitlist/treatment as usual control group, and twenty-one against active control groups for the outcome acceptability/drop-out.

Supplementary table 2 provides data on outcome measures used for extraction and meta-analysis calculations for each individual study.

### Risk of bias

**Fig. 2:**
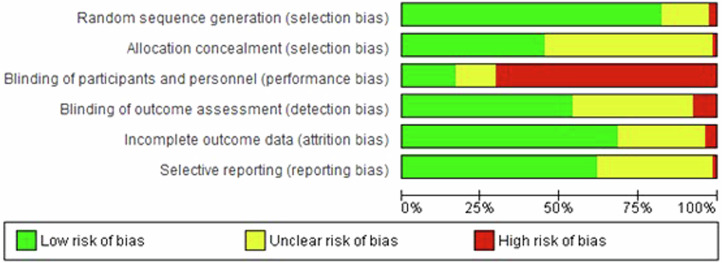
Risk of bias graph. Review authors’ judgements about each risk of bias item presented as percentages across all included studies. Regarding selection bias, 80% of the included studies had a low risk of bias for random allocation, and almost 50% had low risk of bias for allocation concealment. Sixty-five percent of the studies had a high risk of performance bias, due to the inability to blind participants given to the inherent characteristics of the intervention (digital versus waitlist control or standard therapies), which also influenced blinding for outcome assessment especially on self-reported rating scales (52% of the studies had low risk of detection bias). For over 25% of the studies, there was unclear report on how they handled missing data (attrition bias) and 30% had unclear reporting bias (selective outcome reporting) due to the absence of a registered protocol (Fig. 2). These risks of bias are commonly reported by other meta-analyses analyzing digital therapies. Seven out of the 76 studies (9%) were classified as overall low risk of bias (no bias detected). Risk of bias rating for each study is reported in Supplementary Fig. 1.

The inter-rater reliability for the risk of bias assessments showed a Cohen’s kappa of 0.70, indicating good agreement. Regarding selection bias, 80% of the included studies had a low risk of bias for random allocation, and almost 50% had low risk of bias for allocation concealment. Sixty-five percent of the studies had a high risk of performance bias, due to the inability to blind participants given to the inherent characteristics of the intervention (digital versus waitlist control or standard therapies), which also influenced blinding for outcome assessment especially on self-reported rating scales (52% of the studies had low risk of detection bias). For over 25% of the studies, there was unclear report on how they handled missing data (attrition bias) and 30% had unclear reporting bias (selective outcome reporting) due to the absence of a registered protocol (Fig. 2). These risks of bias are commonly reported by other meta-analyses analyzing digital therapies. Seven out of the 76 studies (9%) were classified as overall low risk of bias (no bias detected)^51,58,84,89,98,99,124^. Risk of bias rating for each study is reported in Supplementary Fig. 1.

### Cognition

Data for the outcome cognition was pooled from seventy-one comparisons (*n* = 4345). The random-effects meta-analysis found a small-to-moderate treatment effect of all digital self-guided interventions compared to controls (*g* = −0.51 (95%CI −0.64 to −0.37, *p* < 0.00001)). Heterogeneity between trials was moderate (*I*^2^ = 77%).

**Fig. 3:**
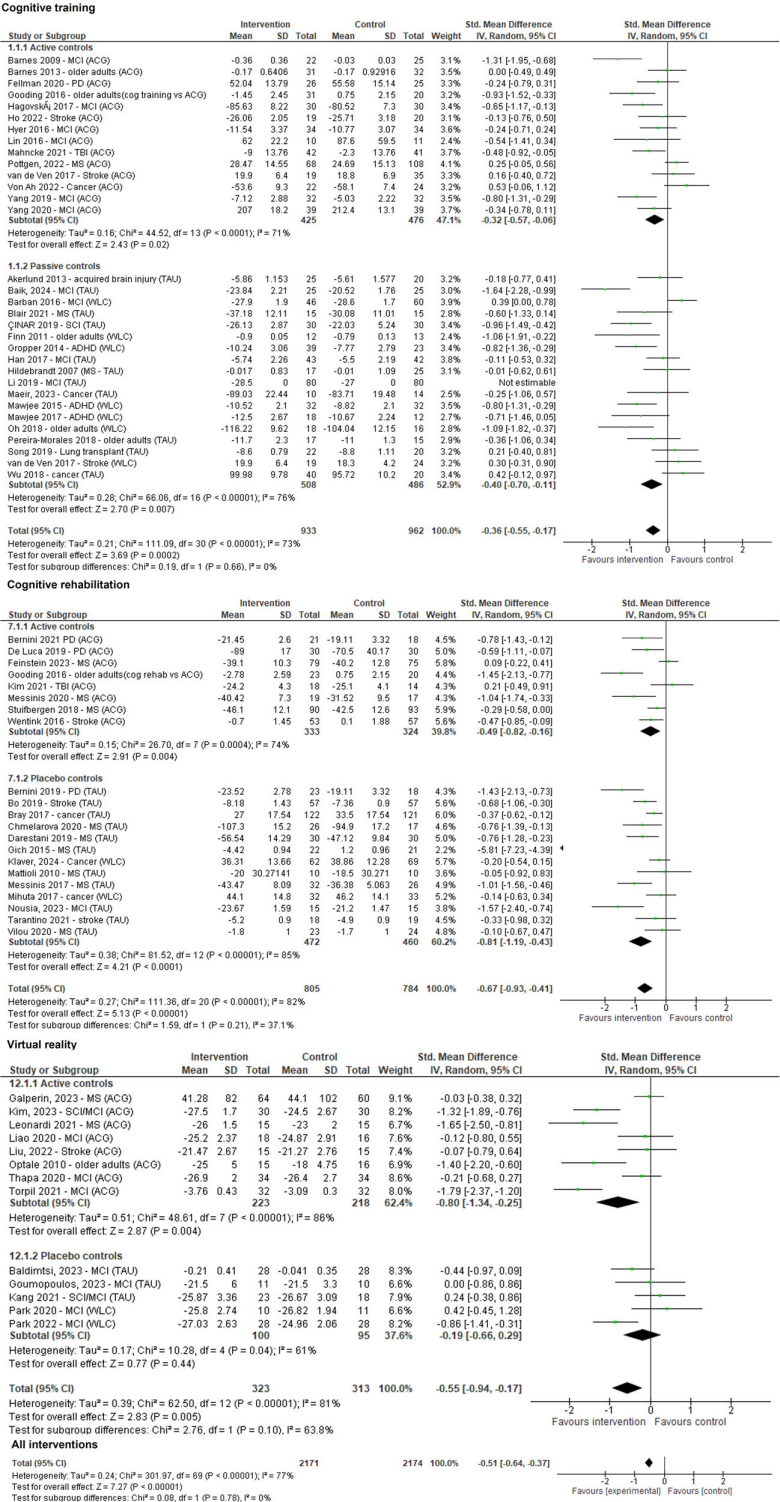
Forest plot for outcome cognition. Plot representing comparison of self-guided digital interventions versus controls (divided by therapeutic framework and active versus non-active controls) for outcome cognition at the end of the intervention. Data was pooled from seventy-one comparisons (*n* = 4345). The random-effects meta-analysis found a small-to-moderate treatment effect of all digital self-guided interventions compared to controls (*g* = –0.51 (95%CI –0.64 to –0.37, *p* < 0.00001)). Heterogeneity between trials was moderate (*I*^2^ = 77%). Analysed independently, a moderate significant effect size was found for both cognitive rehabilitation (*k* = 21, *g* = –0.67, 95% CI –0.93 to –0.41; *Z* = 5.13, *p* < 0.00001) and virtual reality (*k* = 13, *g* = –0.55, 95%CI –0.94 to –0.17; *Z* = 2.83, *p* = 0.005) compared to controls. *I*^2^ statistic identified significant heterogeneity (*I*^2^ = 81%). A small-to-moderate significant effect was found for cognitive training (*k* = 32, *g* = –0.36, 95% CI –0.55 to –0.17; *Z* = 3.69, *p* = 0.0002) and videogames (*k* = 3, *g* = –0.52, 95% CI –-0.86 to –0.18; *Z* = 3.03, *p* = 0.002). The effect of internet-delivered courses on cognition was not significant (*k* = 2, *p* = 0.13). For simplification only cognitive training, cognitive rehabilitation and virtual reality interventions are displayed.

Analysed independently, a moderate significant effect size was found for both cognitive rehabilitation (*k* = 21, *g* = −0.67, 95% CI −0.93 to −0.41; *Z* = 5.13, *p* < 0.00001) and virtual reality (*k* = 13, *g* = −0.55, 95%CI –0.94 to –0.17; *Z* = 2.83, *p* = 0.005) compared to controls. *I*^2^ statistic identified significant heterogeneity (*I*^2^ = 81%). A small-to-moderate significant effect was found for cognitive training (*k* = 32, *g* = –0.36, 95% CI –0.55 to –0.17; *Z* = 3.69, *p* = 0.0002) and videogames (*k* = 3, *g* = –0.52, 95% CI –0.86 to –0.18; *Z* = 3.03, *p* = 0.002). The effect of internet-delivered courses on cognition was not significant (*k* = 2, *p* = 0.13) (Fig. 3 and Table 2).

**Table 2:** Results of quantitative analyses per therapeutic framework and individual outcomes

Outcome	Cognition										
Intervention	*k*	SMD Hedges’s g (95% CI)		Significance of treatment effect	Intervention (*N*)	Control (*N*)			Heterogeneity (Chi^2^; *I*^2^, %)		
Cognitive training											
Active controls	14	−0.32 [−0.57, −0.06]		*Z* = 2.43 (*P* = 0.02)	425	476			44.52; 71%		
Non-active (TAU or WLC)	18	−0.40 [−0.70, −0.11]		*Z* = 2.70 (*P* = 0.0007)	508	486			66.06; 76%		
Total	32	−0.36 [−0.55, −0.17]		*Z* = 3.69 (*P* = 0.0002)	933	962			111.09; 73%		
Subgroup difference: *p* = 0.66											
Cognitive rehabilitation											
Active controls	8	−0.49 [−0.82, −0.16]		*Z* = 2.91 (*P* = 0.004)	333	324			26.70; 74%		
Non-active (TAU or WLC)	13	−0.81 [−1.19, −0.43]		*Z* = 4.21 (*P* < 0.0001)	472	460			81.52; 85%		
Total	21	−0.67 [−0.93, −0.41]		*Z* = 5.13 (*P* < 0.00001)	805	704			111.36; 82%		
Subgroup difference: *p* = 0.21											
Virtual reality											
Active controls	8	−0.80 [−1.34, −0.25]		*Z* = 2.87 (*P* = 0.004)	223	218			48.61; 86%		
Non-active (TAU or WLC)	5	−0.19 [−0.66, 0.29]		*Z* = 0.77 (*P* = 0.44)	100	95			10.28; 61%		
Total	13	−0.55 [−0.94, −0.17]		*Z* = 2.83 (*P* = 0.005)	323	313			62.50; 81%		
Subgroup difference: *p* = 0.10											
Videogames											
Total	3	−0.52 [−0.86, −0.18]		*Z* = 3.03 (*P* = 0.002)	71	70			0.73; 0%		
Internet-delivered cognitive behavioural therapy											
Total	2	−0.74 [−1.68, 0.20]		*Z* = 1.53 (*P* = 0.13)	39	45			4.13; 76%		
All self-guided digital interventions											
Total	71	−0.51 [−0.64, −0.37]		*Z* = 7.27 (*P* < 0.00001)	2171	2174			301.97; 77%		

Outcome	Fatigue/physical health										
Intervention	*k*		SMD Hedges’s g (95% CI)	Significance of treatment effect	Intervention (*N*)		Control (*N*)			Heterogeneity (Chi^2^; *I*^2^, %)	
Cognitive training											
Total	3		0.03 [−0.23, 0.28]	*Z* = 0.21 (*P* = 0.83)	100		148			0.13; 0%	
Cognitive rehabilitation											
Active controls	2		−0.02 [−0.30, 0.27]	*Z* = 0.13 (*P* = 0.90)	98		92			0.45; 0%	
Non-active (TAU or WLC)	2		−0.49 [−0.72, −0.26]	*Z* = 4.22 (*P* < 0.0001)	154		154			0.28; 0%	
Total	4		−0.31 [−0.61, −0.00]	*Z* = 1.97 (*P* = 0.05)	252		246			7.15; 58%	
Subgroup difference: *p* = 0.01											
Virtual reality											
Total	1		−0.80 [−1.17, −0.43]	*Z* = 4.40 (*P* < 0.0001)	64		**60**			N/A	
All self-guided digital interventions											
Total	8		−0.27 [−0.53, −0.02]	*Z* = 2.13 (*P* = 0.03)	416		454			20.74; 66%	

Outcome	Activities of daily living										
Intervention	*k*		SMD Hedges’s g (95% CI)	Significance of treatment effect	Intervention (*N*)		Control (*N*)			Heterogeneity (Chi^2^; *I*^2^, %)	
Cognitive training											
Active controls	7		−0.03 [−0.21, 0.15]	*Z* = 0.30 (*P* = 0.76)	225		268			1.96; 0%	
Non-active (TAU or WLC)	2		−0.25 [−0.69, 0.18]	*Z* = 1.16 (*P* = 0.25)	40		44			0.21; 0%	
Total	9		−0.06 [−0.23, 0.10]	*Z* = 0.72 (*P* = 0.47)	265		312			1.71; 0%	
Subgroup difference: *p* = 0.34											
Cognitive rehabilitation											
Active controls	2		0.12 [−0.17, 0.41]	*Z* = 0.80 (*P* = 0.42)	97		89			0.35; 0%	
Non-active (TAU or WLC)	3		−0.08 [−0.33, 0.16]	*Z* = 0.65 (*P* = 0.52)	120		137			1.44; 0%	
Total	5		0.00 [−0.18, 0.19]	*Z* = 0.02 (*P* = 0.98)	217		226			2.85; 0%	
Subgroup difference: *p* = 0.30											
Virtual reality											
Total	5		−0.29 [−0.57, −0.02]	*Z* = 2.09 (*P* = 0.04)	123		117			4.30; 7%	
Internet-delivered cognitive behavioural therapy											
Total	2		−0.28 [−0.71, 0.15]	*Z* = 1.28 (*P* = 0.20)	39		45			0.09; 0%	
All self-guided digital interventions											
Total	21		−0.09 [−0.20, 0.02]	*Z* = 1.67 (*P* = 0.09)	644		700			14.16; 0%	

Outcome	Mental health (depression and anxiety)										
Intervention	*k*		SMD Hedges’s g (95% CI)	Significance of treatment effect	Intervention (*N*)		Control (*N*)			Heterogeneity (Chi^2^; *I*^2^, %)	
Cognitive training											
Active controls	5		−0.18 [−0.51, 0.16]	*Z* = 1.02 (*P* = 0.31)	184		212			9.71; 59%	
Non-active (TAU or WLC)	7		−0.45 [−0.96, 0.07]	*Z* = 1.69 (*P* = 0.09)	173		173			32.06; 81%	
Total	12		−0.34 [−0.65, −0.02]	*Z* = 2.10 (*P* = 0.04)	264		252			46.04; 76%	
Subgroup difference: *p* = 0.39											
Cognitive rehabilitation											
Active controls	5		−0.28 [−0.58, 0.02]	*Z* = 1.80 (*P* = 0.07)	241		235			9.21; 57%	
Non-active (TAU or WLC)	4		−1.19 [−2.21, −0.17]	*Z* = 2.29 (*P* = 0.02)	177		170			32.56; 91%	
Total	9		−0.64 [−1.04, −0.23]	*Z* = 3.07 (*P* = 0.002)	418		405			54.50; 85%	
Subgroup difference: *p* = 0.09											
Virtual reality											
Active controls	4		−0.52 [−0.78, −0.27]	*Z* = 4.00 (*P* < 0.0001)	124		121			2.96; 0%	
Non-active (TAU or WLC)	3		0.11 [−0.33, 0.54]	*Z* = 0.48 (*P* = 0.63)	44		39			0.55; 0%	
Total	7		−0.36 [−0.66, −0.06]	*Z* = 2.37 (*P* = 0.02)	168		160			9.50; 37%	
Subgroup difference: *p* = 0.01											
Internet-delivered cognitive behavioural therapy											
Total	2		−0.18 [−0.61, 0.25]	*Z* = 0.81 (*P* = 0.42)	39		45			0.10; 0%	
All self-guided digital interventions											
Total	30		−0.41 [−0.60, −0.22]	*Z* = 4.20 (*P* < 0.0001)	982		995			115.81; 75%	

Outcome	Quality of life										
Intervention	*k*		SMD Hedges’s g (95% CI)	Significance of treatment effect	Intervention (*N*)		Control (*N*)			Heterogeneity (Chi^2^; *I*^2^, %)	
Cognitive training											
Active controls	4		−0.51 [−1.28, 0.25]	*Z* = 1.32 (*P* = 0.19)	162		203			33.12; 91%	
Non-active (TAU or WLC)	4		−0.05 [−0.37, 0.28]	*Z* = 0.28 (*P* = 0.78)	82		74			1.03; 0%	
Total	8		−0.29 [−0.71, 0.14]	*Z* = 1.37 (*P* = 0.17)	244		277			35.18; 80%	
Subgroup difference: *p* = 0.27											
Cognitive rehabilitation											
Active controls	3		−0.02 [−0.25, 0.21]	*Z* = 0.17 (*P* = 0.86)	151		149			1.18; 0%	
Non-active (TAU or WLC)	5		−0.12 [−0.29, 0.05]	*Z* = 1.38 (*P* = 0.17)	257		267			3.30; 0%	
Total	8		−0.08 [−0.22, 0.05]	*Z* = 1.21 (*P* = 0.23)	408		416			4.96; 0%	
Subgroup difference: *p* = 0.49											
Virtual reality											
Total	3		−0.38 [−1.14, 0.37]	*Z* = 1.00 (*P* = 0.32)	102		93			10.26; 81%	
Videogames											
Total	2		−0.08 [−0.51, 0.36]	*Z* = 0.34 (*P* = 0.73)	41		40			0.09; 0%	
Internet-delivered cognitive behavioural therapy											
Total	1		−0.40 [−1.12, 0.32]	*Z* = 1.08 (*P* = 0.28)	13		18			N/A	
All self-guided digital interventions											
Total	22		−0.17 [−0.34, −0.00]	*Z* = 2.01 (*P* = 0.04)	808		844			52.90; 60%	

Outcome	Acceptability: treatment dropout										
Intervention	*k*		Odds Ratio (95% CI)	Significance of treatment effect	Intervention			Control			Heterogeneity (Chi^2^; *I*^2^, %)
Cognitive training											
Active controls	12		1.23 [0.80, 1.88]	*Z* = 0.93 (*P* = 0.35)	64/360			62/422			12.42; 11%
Non-active (TAU or WLC)	13		1.53 [0.70, 3.38]	*Z* = 1.06 (*P* = 0.29)	65/399			44/330			28.05; 54%
Total	24		1.25 [0.82, 1.90]	*Z* = 1.04 (*P* = 0.30)	129/759			106/752			40.46; 38%
Subgroup difference: *p* = 0.62											
Cognitive rehabilitation											
Active controls	5		1.19 [0.36, 3.90]	*Z* = 0.29 (*P* = 0.77)	21/261			17/260			7.38; 46%
Non-active (TAU or WLC)	8		1.67 [1.16, 2.42]	*Z* = 2.74 (*P* = 0.006)	99/407			63/394			5.32; 0%
Total	13		1.59 [1.10, 2.28]	*Z* = 2.49 (*P* = 0.01)	120/668			80/654			13.29; 10%
Subgroup difference: *p* = 0.59											
Virtual reality											
Active controls	5		0.74 [0.24, 2.30]	*Z* = 0.51 (*P* = 0.61)	6/117			8/118			1.11; 0%
Non-active (TAU or WLC)	2		1.12 [0.19, 6.45]	*Z* = 0.12 (*P* = 0.90)	3/34			2/28			0.48; 0%
Total	7		0.84 [0.32, 2.16]	*Z* = 0.37 (*P* = 0.71)	9/151			10/146			1.73; 0%
Subgroup difference: *p* = 0.70											
Videogames											
Total	2		0.30 [0.01, 7.81]	*Z* = 0.73 (*P* = 0.47)	0/48			1/47			N/A
Internet-delivered cognitive behavioural therapy											
Total	2		1.57 [0.38, 6.53]	*Z* = 0.62 (*P* = 0.53)	5/42			4/46			0.63; 0%
All self-guided digital interventions											
Total	48		1.34 [1.03, 1.74]	*Z* = 2.19 (*P* = 0.03)	262/1648			204/1646			56.57; 20%

*TAU* treatment as usual, *WLC* waitlist control, *SMD* standardized mean difference/effect estimate.

### Fatigue/physical health

**Fig. 4:**
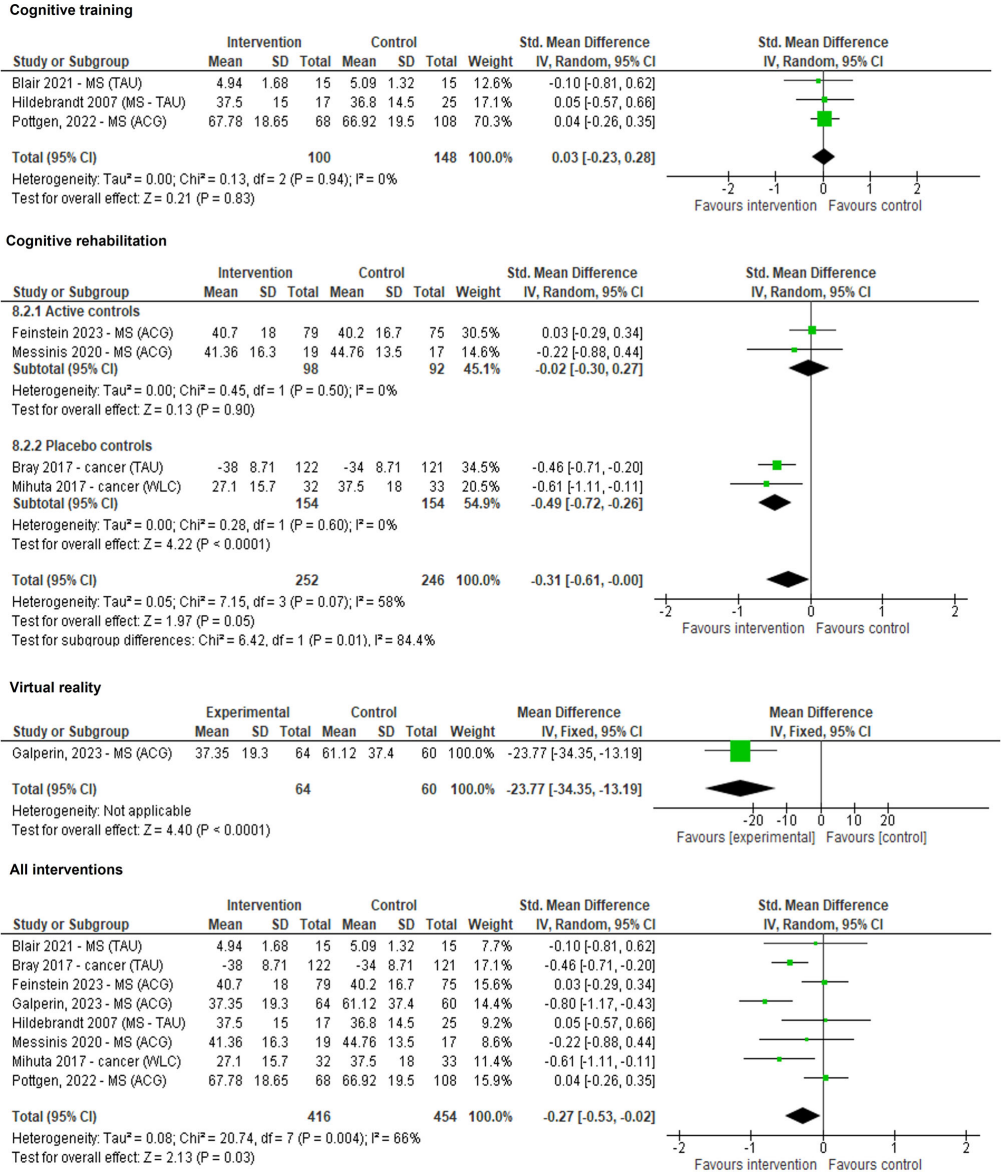
Forest plot for outcome fatigue/physical health. Plot representing comparison of self-guided digital interventions versus controls (divided by therapeutic framework and active versus non-active controls) for outcome fatigue/physical health at the end of the intervention. Data was pooled from eight comparisons (*n* = 870). Only cognitive training (*k* = 3), cognitive rehabilitation (*k* = 4), and virtual reality (k = 1) reported data on fatigue/physical health outcomes. The random-effects meta-analysis found only a marginally significant effect of all self-help interventions compared to controls (*g* = −0.27 (95%CI −0.53 to −0.02; *p* = 0.03, *I*^2^ = 66%).

Data for the outcome fatigue/physical health was pooled from eight comparisons (*n* = 870). Only cognitive training (*k* = 3)^80,91,95^, cognitive rehabilitation (*k* = 4)^51,89,105,106^, and virtual reality (*k* = 1)^96^ reported data on fatigue/physical health outcomes. The random-effects meta-analysis found only a marginally significant effect of all self-help interventions compared to controls (*g* = −0.27 (95%CI −0.53 to −0.02; *p* = 0.03, *I*^2^ = 66%) (Fig. 4 and Table 2).

### Activities of daily living

**Fig. 5:**
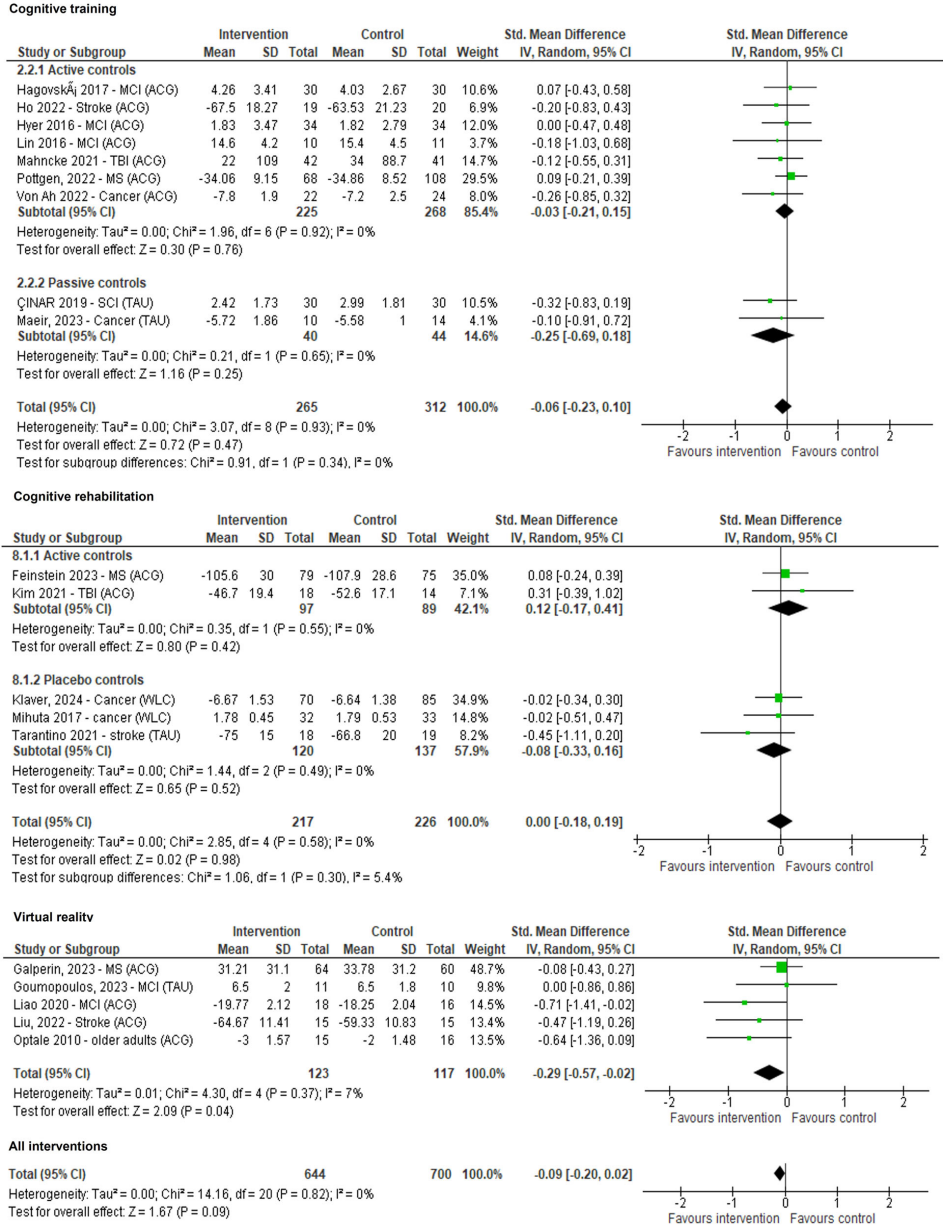
Forest plot for outcome performance in activities of daily living. Plot representing comparison of self-guided digital interventions versus controls (divided by therapeutic framework and active versus non-active controls) for outcome performance in activities of daily living at the end of the intervention. Data was pooled from twenty-one comparisons (*n* = 1344). The effect of all self-help interventions was not significant (*p* = 0.09). From all therapeutic approaches, only virtual reality provided a marginal significant effect in improving performance of activities of daily living relative to controls (*k* = 5, *g* = −0.29, 95% CI −0.57 to −0.02; *Z* = 2.09, *p* = 0.04). Effects of self-guided cognitive training, cognitive rehabilitation and internet-delivered cognitive behavioural were not significant. For simplification only cognitive training, cognitive rehabilitation and virtual reality interventions are displayed.

Data for the outcome activities of daily living was pooled from twenty-one comparisons (*n* = 1344). The effect of all self-help interventions was not significant (*p* = 0.09). From all therapeutic approaches, only virtual reality provided a marginal significant effect in improving performance of activities of daily living relative to controls (*k* = 5, *g* = −0.29, 95% CI −0.57 to −0.02; *Z* = 2.09, *p* = 0.04). Effects of self-guided cognitive training, cognitive rehabilitation and internet-delivered cognitive behavioural were not significant (Fig. 5 and Table 2).

### Mental health

Data on mental health outcomes was pooled from thirty comparisons (*n* = 1977). The random-effects meta-analysis found a small significant treatment effect of all self-guided interventions compared to controls (*g* = −0.41 (95%CI −0.60 to −0.22; *z* = 4.20; *p* < 0.0001)). Heterogeneity was high (*I*^2^ = 75%).

**Fig. 6:**
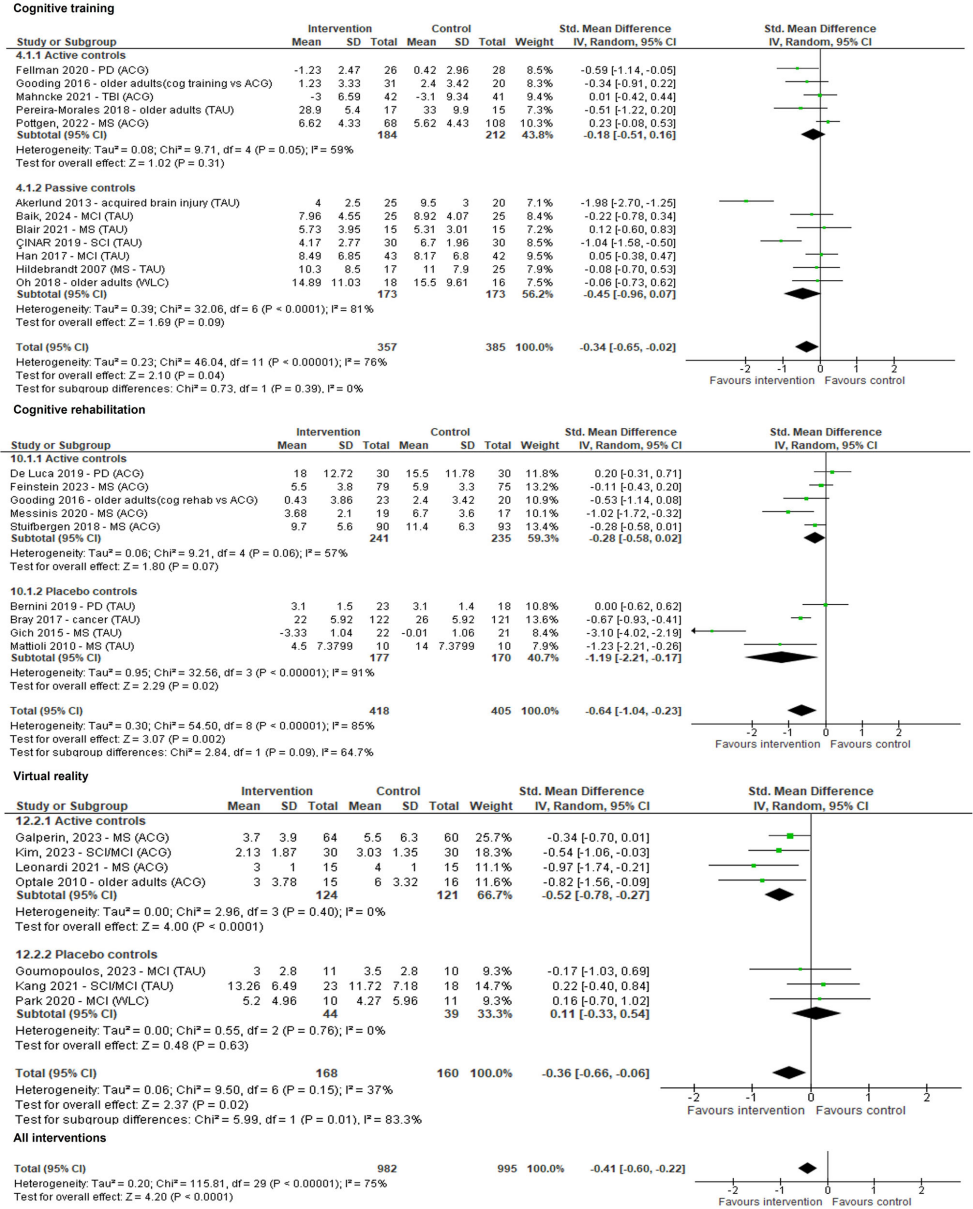
Forest plot for outcome mental health. Plot representing comparison of self-guided digital interventions versus controls (divided by therapeutic framework and active versus non-active controls) for outcome mental health at the end of the intervention. Data was pooled from thirty comparisons (*n* = 1977). The random-effects meta-analysis found a small significant treatment effect of all self-guided interventions compared to controls (*g* = −0.41 (95% CI −0.60 to −0.22; *z* = 4.20; *p* < 0.0001)). Self-guided digital cognitive training interventions provided only a marginal significant treatment effect (*k* = 12, *g* = −0.34, 95% CI −0.65 to −0.02; *z* = 2.10; *p* = 0.04; *I*^2^ = 76%), while self-guided digital cognitive rehabilitation provided a moderate significant treatment effect (*k* = 9, *g* = −0.64, 95% CI −1.04 to −0.23; *Z* = 3.07, *p* = 0.002; *I*^2 ^= 85%). Virtual reality provided a small significant treatment effect versus controls (*k* = 7, *g* = −0.36, 95% CI −0.66 to −0.06; *z* = 2.37; *p* = 0.02; *I*^2^ = 37%) that was more pronounced against active control groups. The effects of internet-delivered cognitive behavioural therapy on mental health outcomes were non-significant (*k* = 2, *g* = −0.18, 95% CI −0.61 to 0.25; *Z* = 0.81, *P* = 0.42; *I*^2^ = 0%), while videogames did not provide data for meta-analysis. For simplification only cognitive training, cognitive rehabilitation and virtual reality interventions are displayed.

Self-guided digital cognitive training interventions provided only a marginal significant treatment effect (*k* = 12, *g* = −0.34, 95% CI −0.65 to −0.02; *z* = 2.10; *p* = 0.04; *I*^2^ = 76%), while self-guided digital cognitive rehabilitation provided a moderate significant treatment effect (*k* = 9, *g* = −0.64, 95% CI −1.04 to −0.23; *Z* = 3.07, *p* = 0.002; *I*^2^ = 85%). Virtual reality provided a small significant treatment effect versus controls (*k* = 7, *g* = −0.36, 95% CI −0.66 to −0.06; *z* = 2.37; *p* = 0.02; *I*^2^ = 37%) that was more pronounced against active control groups. The effects of internet-delivered cognitive behavioural therapy on mental health outcomes were non-significant (*k* = 2, *g* = −0.18, 95% CI −0.61 to 0.25; *Z* = 0.81, *P* = 0.42; *I*^2^ = 0%), while videogames did not provide data for meta-analysis (Fig. 6).

### Quality of life

**Fig. 7:**
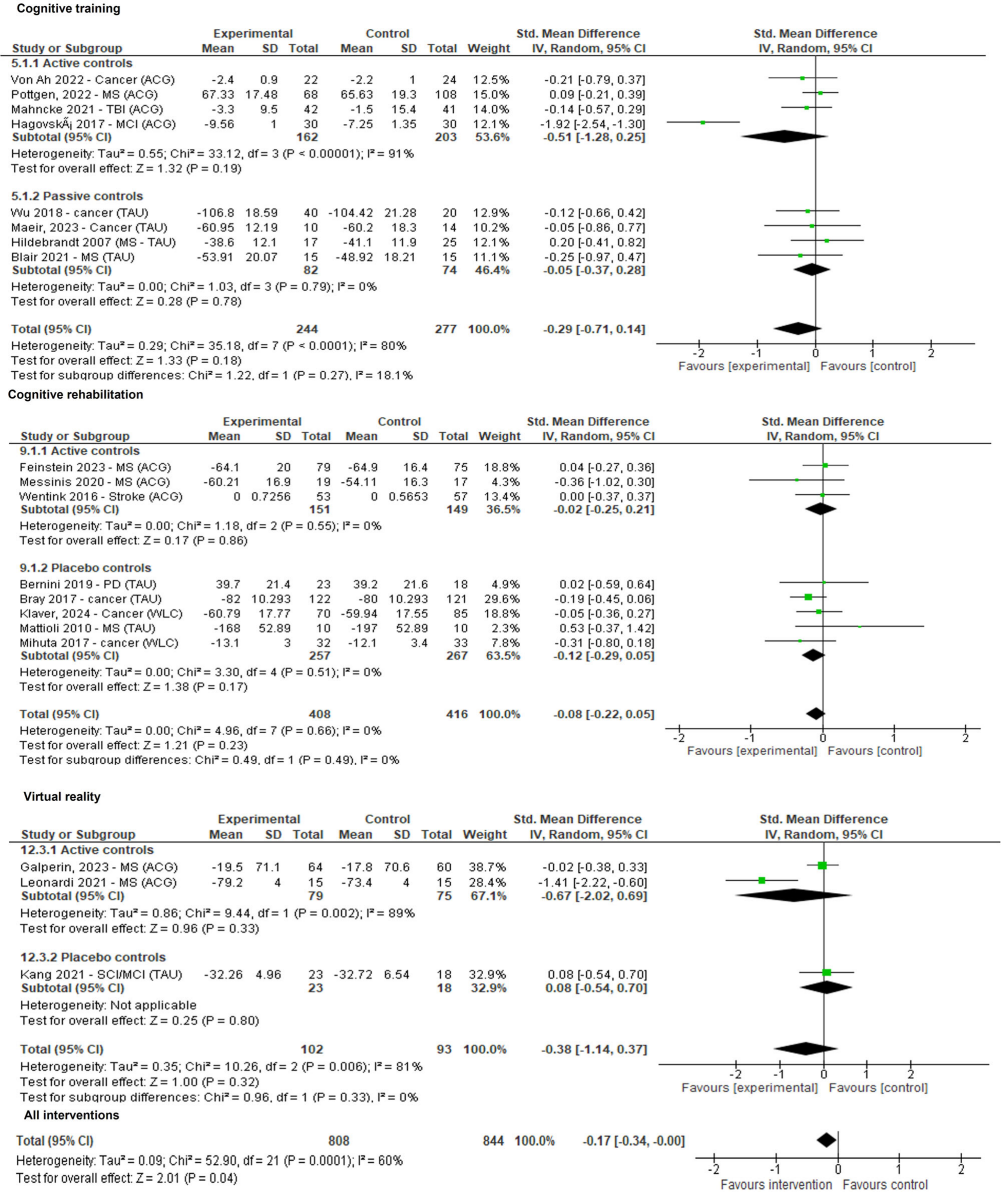
Forest plot for outcome quality of life. Plot representing comparison of self-guided digital interventions versus controls (divided by therapeutic framework and active versus non-active controls) for outcome quality of life at the end of the intervention. The pooled effect of all interventions (twenty-two comparisons, *n* = 1652) was only marginally significant (*g* = −0.17 (95% CI −0.34 to −0.00; *p* = 0.04; *I*^2^ = 60%). When analysed independently, the effects of all different intervention frameworks were non-significant. For simplification only cognitive training, cognitive rehabilitation and virtual reality interventions are displayed.

All intervention frameworks targeting cognition included trials assessing quality of life (twenty-two comparisons, *n* = 1652). The pooled effect of all interventions was only marginally significant (*g* = −0.17 (95%CI −0.34 to −0.00; *p* = 0.04; *I*^2^ = 60%). When analysed independently, the effects of all different intervention frameworks were non-significant (Fig. 7).

### Acceptability: treatment dropout

Data pooled from 48 comparisons (*n* = 3294) suggests that being on the self-guided digital arm might slightly increase the odds of dropping the study versus the control arm (OR = 1.34, 95% CI 1.03−1.74; *Z* = 2.19, *p* = 0.03, *I*^2^ = 20%).

### Subgroup analyses

Due to the heterogeneity between trials we conducted subgroup analyses. Table 3 provides data for subgroup analysis examining moderators of treatment effects on the outcome cognition. Regarding trial design characteristics, there was no significant relationship between control groups (active vs treatment as usual or waitlist controls) (*p* = 0.78), with both comparisons showing small-to-moderate significant treatment effects of all self-guided digital interventions on cognition.

We analyzed if different patient characteristics influenced the treatment effects of all self-help digital interventions. Overall, in descending order, studies focusing on ADHD (*k* = 5), Parkinson’s disease (*k* = 4), MCI/subjective cognitive symptoms (*k* = 28) and multiple sclerosis (*k* = 16), ADHD (*k* = 5), and exhibited significantly greater treatment effects (moderate to large effects), while no benefit was found post-stroke (*k* = 7), in cancer survivors (*k* = 7), post-traumatic brain injury (*k* = 3) and post-lung transplant (*k* = 1), and the difference between groups was statistically significant (*p* < 0.00001).

Interestingly, trials using virtual reality (*k* = 13) and videogames (*k* = 3) digital interventions appear to exhibit greater treatment benefits for cognitive symptoms in comparison to the other formats, but the difference between groups regarding mode of delivery was not statistically significant (*p* = 0.56) (supplementary Fig. 2).

There was no significant difference in the efficacy of interventions that were 6 weeks or shorter compared to interventions with a duration of >6 weeks (*p* = 0.32), nor between RCTs with high versus low risk of bias (*p* = 0.17) (Table 3).

**Table 3:** Subgroup analysis of self-help digital interventions on outcome cognition

Moderator	*k*	SMD Hedges’s g (95% CI)	Significance of treatment effect	Intervention (*N*)	Control (*N*)	Weight	Heterogeneity (Chi^2^; *I*^2^, %)
Control group							
Active controls	30	−0.48 [−0.68, −0.29]	*Z* = 4.80 (*P* < 0.00001)	981	1018	44.3%	128.44; 77%
Non−active (TAU or WLC)	41	−0.52 [−0.71, −0.33]	*Z* = 5.40 (*P* < 0.00001)	1190	1156	55.7%	171.94; 77%
Total	71	−0.51 [−0.64, −0.37]	*Z* = 7.27 (*P* < 0.00001)	2171	2174	100%	236.30; 75%
Subgroup difference: *p* = 0.78							
Population							
MCI/SCI	28	−0.66 [−0.90, −0.41]	*Z* = 5.33 (*P* < 0.00001)	767	762	37.8%	117.70; 78%
Multiple sclerosis	16	−0.63 [−0.98, −0.28]	*Z* = 3.56 (*P* = 0.0004)	558	583	22.7%	108.74; 86%
Traumatic brain injury	3	−0.23 [−0.61, 0.15]	*Z* = 1.17 (*P* = 0.24)	85	75	4.4%	2.78; 28%
Parkinson’s disease	4	−0.72 [−1.18, −0.26]	*Z* = 3.08 (*P* = 0.002)	100	91	5.7%	7.06; 58%
Post−stroke symptoms	7	−0.23 [−0.51, 0.06]	*Z* = 1.58 (*P* = 0.11)	200	227	10.4%	11.73; 49%
Cancer survivors	7	−0.08 [−0.34, 0.17]	*Z* = 0.64 (*P* = 0.52)	311	304	10.7%	12.71; 53%
ADHD	5	−0.79 [−1.07, −0.52]	*Z* = 5.66 (*P* < 0.00001)	128	112	7.0%	4.19; 5%
Lung transplant	1	0.21 [−0.40, 0.81]	*Z* = 0.66 (*P* = 0.51)	22	20	1.7%	N/A
Total	71	−0.51 [−0.64, −0.37]	*Z* = 7.27 (*P* < 0.00001)	2171	2174	100%	301.97; 77%
Subgroup difference: *p* = 0.0003							
Delivery format							
Computerized	50	−0.46 [−0.64, −0.29]	*Z* = 5.19 (*P* < 0.00001)	1634	1524	69.1%	206.34; 80%
App	3	−0.77 [−1.53, 0.00]	*Z* = 1.95 (*P* = 0.05)	87	85	4.8%	10.83; 82%
Virtual reality	13	−0.55 [−0.94, −0.17]	*Z* = 2.83 (*P* = 0.005)	323	313	19.7%	62.50; 81%
Videogames	3	−0.52 [−0.86, −0.18]	*Z* = 3.03 (*P* = 0.002)	71	70	4.8%	0.73; 0%
Compact disc (CD)	2	−0.01 [−0.62, 0.61]	*Z* = 0.02 (*P* = 0.98)	56	25	1.6%	N/A
Total	71	−0.49 [−0.64, −0.34]	*Z* = 6.58 (*P* < 0.00001)	2171	2017	100%	287.21; 78%
Subgroup difference: *p* = 0.56							
Treatment duration							
≤6 weeks	26	−0.43 [−0.61, −0.24]	*Z* = 7.01 (*P* < 0.00001)	1925	1915	90.5%	284.38; 78%
>6 weeks	45	−0.56 [−0.75, −0.37]	*Z* = 5.81 (*P* < 0.00001)	1430	1427	62.2%	227.62; 81%
Total	71	−0.51 [−0.64, −0.37]	*Z* = 6.78 (*P* < 0.00001)	1766	1723	100%	301.97; 77%
Subgroup difference: *p* = 0.32							
Risk of bias							
High risk	65	−0.53 [−0.68, −0.38]	*Z* = 6.64 (*P* < 0.00001)	1665	1594	90.5%	283.90; 80%
Low risk	6	−0.29 [−0.60, 0.02]	*Z* = 1.83 (*P* = 0.07)	246	259	9.5%	13.81; 64%
Total	71	−0.51 [−0.64, −0.37]	*Z* = 7.27 (*P* < 0.00001)	2171	2174	100%	301.97; 77%
Subgroup difference: *p* = 0.17							

*SMD* standardized mean difference/effect estimate.

### Publication bias

For the outcome cognition, although visual inspection of the funnel plot did not suggest publication bias, Egger’s test was significant for cognitive training (*p* < 0.0001) and cognitive rehabilitation interventions (*p* = 0.003), and internet CBT-based programmes (*p* < 0.0001). For the outcome mental health, Egger’s test suggests that unpublished studies with opposing effects may eventually exist for cognitive training (*p* = 0.04).

No significant publication bias was found on most comparisons for activities of daily living, quality of life and physical health/fatigue outcomes among the pooled studies (supplementary Figs. 3–7).

## Discussion

This systematic review and meta-analysis investigating the efficacy of self-help digital interventions for patients with transdiagnostic cognitive symptoms in patients without dementia found 76 eligible trials. Trials privileged more established therapeutic frameworks such as computerized cognitive training and rehabilitation, which have been ‘in the market’ for longer time, with fewer trials focusing on virtual reality, videogames and internet-based courses. Unexpectedly, despite the availability of more than 350,000 health-related mobile apps for download, we found only three trials evaluating apps as a delivery format. These findings suggest that most commercially digital self-guided interventions for cognitive symptoms remain untested in RCTs.

Self-guided digital cognitive training, cognitive rehabilitation and virtual reality (against active controls) were the most effective interventions at improving cognition, with a pooled small-to-moderate treatment effect for all interventions. Our study also suggests a small benefit of digital self-guided interventions for mental health, with greater benefits reported with cognitive rehabilitation and virtual reality interventions. These effect sizes are in line with previous evidence for other cognitive interventions in SCD^21^ and mental health interventions^24,26^. The present study identified several important shortcomings in this field as data on fatigue, activities of daily living, quality of life, and other potentially relevant measures including insomnia and frailty, were often not assessed and/or reported. We found little evidence to support a marginal improvement on fatigue and quality of life provided by standalone digital self-guided cognitive interventions, and this remains to be confirmed in future studies. Overall, we did not find evidence to support an improvement in performance of activities of daily living, albeit virtual reality figured as the most promising therapeutic approach in this regard. While data for these non-cognitive outcomes was pooled from a reduced number of studies, the overall lack of treatment effects beyond cognition and mental health may also be partially explained by the nature of most of these interventions. Cognitive training and cognitive rehabilitation focus on specific cognitive domains and the benefits may not translate into improvement in other non-trained cognitive domains and broader functional outcomes^128^. Further, self-perception of cognitive difficulties can lead to increased distress, fear of dementia, anxiety and depression, and reduction of quality of life, all of which can aggravate cognitive symptoms, but traditionally these are not targeted by cognitive interventions. While internet-delivered psychotherapy programmes have been tested in various mental^26^ and neurological conditions (eg, insomnia, fatigue, and pain)^129–131^, with small-to moderate significant treatment effects, we only found two trials evaluating self-help internet-delivered CBT-based courses for cognitive symptoms, both for people with ADHD. Novel therapeutic approaches for cognition including cognitive restructuring, stress reduction and self-regulation techniques promoting attentional re-focusing, more adaptive and realistic attitudes toward memory performance^9,132,133^ may be beneficial, but research is warranted to identify the key elements by which these programmes may improve cognitive and related functions. Notably, evidence exists that education programmes and cognitive restructuring are up to five times more effective than cognitive rehabilitation and memory training in reducing post-concussion cognitive symptoms^134^ and subjective memory symptoms^21,133^ respectively. Similarly, a group and therapist-assisted cognitive behavioural programme provided benefits for quality of life and attention in patients with epilepsy^135^.

Results from our subgroup analyses suggest that virtual reality software and videogames may provide greater cognitive benefits in comparison to other delivery formats, although the difference was not significant. These results are limited by the reduced number of studies limiting the power of this analysis, and high heterogeneity, but they deserve further consideration. Virtual reality elicits virtual sensations through the creation of a model simulation of virtual body and surroundings triggering immediate responses via real-time feedback mechanisms. Studies in MCI have found potential benefits of virtual reality for cognition and daily life functions^136^. It remains untested whether virtual reality benefits could be potentiated in combination with other therapeutic frameworks including psychotherapy, as explored in other neurological disorders^137,138^. Similarly, controversy exists regarding whether immersive technologies provide higher benefits than non-immersive programmes, particularly for non-cognitive outcomes, although a systematic review suggested that semi-immersive is more effective than immersive software in improving cognitive flexibility, and non-immersive virtual reality can significantly improve global cognitive function, attention, short-term memory, and cognitive flexibility^139^. The effect size for apps was also moderate but given that only three trials were included, this was not statistically significant. Thus, preliminary evidence suggests that newer delivery formats may offer promising treatment benefits when compared to computerized interventions despite much larger evidence for the latter, and this remains to be explored in future studies. Moreover, one limitation is that most of these other format interventions are currently not available in routine care outside of research projects.

Our subgroup analysis revealed additional information on the potential differences and sources of heterogeneity in our results. Overall, all interventions provided greater benefits in reducing cognitive symptoms in MCI/subjective cognitive symptoms, multiple sclerosis, ADHD and Parkinson’s disease populations. However, the same was not replicated in trials recruiting patients that had suffered traumatic brain injury, cancer survivors and post-stroke symptoms, for which these interventions were largely ineffective. This is in line with previous studies^9,42^ and we hypothesize that it is at least partially explained by the fact that digital interventions in stroke and TBI are used in a more acute setting which may determine an improvement in both treatment groups due to the natural history of the disease, and partially because structural brain lesions often contribute to the cognitive symptoms in these populations.

We did not find a difference in treatment effects when comparing interventions to active controls versus treatment as-usual/waitlist control groups. Also, longer and shorter interventions performed similarly, supporting the idea that longer treatment exposure does not necessarily translate to higher treatment benefits, even in standalone self-help digital therapies^140^. While treatment duration is unlikely to be standardized, in our review we found a median number of sessions per week was 3, with a median of 45 min per session. As data shows that 30-day retention rates with remote interventions are generally low^35^, perhaps shorter interventions can be applied with a focus on upgrading patient engagement and retention, both of which are key determinants of treatment success.

It seems intuitive but it remains to be tested whether combining different approaches from the different studies, plus information on the nature of symptoms and self-management strategies, may potentiate treatment benefits for individuals with transdisciplinary cognitive symptoms. Tentatively, combinations of digital interventions with other non-pharmacological interventions, including physical exercise and mindfulness, have been explored^51,141–143^.

We followed strict inclusion criteria, to include only RCTs analyzing self-guided interventions, as we wished to explore if these interventions are suitable for scalability and an effective alternative to therapist-driven interventions in stepped models of care. Yet, several limitations should be considered. Heterogeneity between trials might decrease the confidence in these results for some of the comparisons made, especially those with a limited number of studies available. Most trials were conducted in high-income countries, they were all published in English, and grey literature was not included which may limit the comprehensiveness of our meta-analysis^144^. In practice, boundaries between therapeutic frameworks could be less clear-cut (e.g. video-sharing principles with cognitive training), and we grouped the interventions according to their main distinguishing element, and classification attributed by the authors of each study. Overall risk of bias for most trials was considered high, due to high or unclear risk in some domains, mainly due to a lack of study pre-registration and failure to blind patients in the treatment arm due to the digital nature of the intervention. Minimal information was available on treatment programmes, usability and participant engagement within RCTs, so heterogeneity inter-interventions and efficacy could not be explored in a content and feature-focused manner. Further, although the inclusion of a range of different outcomes would allow for assessment of interventions in a more clinically meaningful way, only a few studies assessed and/or reported physical health/fatigue, activities of daily living, mental health and quality of life outcomes, despite a known poor correlation between objective memory functioning and subjective distress caused by cognitive symptoms^145,146^. Plus, heterogeneous measures were used by the different studies, and it is likely that some outcome measures are interrelated (e.g. quality of life measures are influenced and address one’s mental health and physical health/fatigue) despite their categorization. Despite an extensive search, the number of identified RCTs for treatment approaches and formats other than computerized cognitive training and rehabilitation was small, with a trend for newer RCTs to focus on novel formats such as virtual reality. Publication bias cannot be excluded. A number of the meta-analyses included only a small number of studies and were not adequately powered to detect clinically relevant differences between these interventions and controls. Additionally, criteria for evaluation of cognitive symptoms or impairment and inclusion in the studies varied in individual RCTs. However, we decided to be over-inclusive as many of these patients will have symptoms driven by similar mechanisms, present to memory services, and currently lack therapeutic options.

We did not pool long-term follow-up outcome data because these were variable and only reported by a minority of studies. Thus, no conclusions about the long-term efficacy were drawn from the present study. It is possible that implementation of learned strategies for a certain period of time is needed before clinical benefits are detectable. It is also noted that post-intervention measurement times also varied between studies. Likewise, potential adverse effects of these technologies remain largely unexplored due to underreporting. However, pooled data suggests a possible increase in dropout in the intervention arm (OR = 1.34 (1.03–1.74). Finally, this study did not seek to explore the effect of these interventions to prevent progression to dementia. Although the majority of patients with SCD does not progress to dementia^147^, it is possible that some of these studies included patients with prodromal dementia.

In conclusion, findings of this systematic review and meta-analysis indicate that cognitive and mental health symptoms may be amenable to novel self-guided digital transdiagnostic interventions for cognitive symptoms, in at least a subset of patients. Benefits may potentially translate to small improvements in fatigue and quality of life outcomes, although this remains unclear. Newer methods like virtual reality appear promising to improve functional domains, but further research under routine conditions is needed to propel the field forward and ensure the delivery of evidence-based care to patients experiencing cognitive symptoms. Despite the prolific number of cognitive apps, the field lacks evidence-based treatments, trailing behind other areas like mental health and other chronic conditions^24,25^. Potential barriers for the implementation of these technologies, besides those reported by healthcare professionals^148^, include access to systems, costs, regulations, academic-industry partnerships and patient involvement and satisfaction, and also remain largely unexplored. Future studies will help identify which groups are most likely to benefit from self-guided interventions and which format to use, including considerations of user cultural and educational, age and neural inter-individual differences^149^.

## Methods

This review was registered on OSF (10.17605/OSF.IO/V6T3K). It was conducted in accordance with the Preferred Reporting Items for Systematic Reviews and Meta-Analyses (PRISMA) statement guideline^150^ and the Cochrane Handbook for Systematic Reviews of Interventions^151^ (Supplementary Table 4).

### Study eligibility

Our eligibility criteria included 1) RCTs only, investigating the effects of 2) standalone digital (computer, virtual reality or mobile-based) interventions that 3) were designed with the intent of reducing cognitive symptoms, and are 4) self-guided (patients independently engage with the intervention and contact with therapists is only allowed at the start and/or completion of the intervention, or for sporadic telephone support, but not for the delivery of the intervention^152^.

The trials must have included an adult (≥18 years) population with cognitive symptoms, compared to a control condition either inactive (treatment as usual or waitlist) or active (sham or traditional face-to-face therapy), and a minimum sample size of 10 patients in each group. No minimum dose was set. Only articles published in peer-reviewed journals were considered.

We excluded quasi-randomized studies, and those carried out in populations with dementia or a major psychiatric disorder (e.g. schizophrenia). Interventions requiring therapist guidance (e.g. full psychotherapy, major support, or group intervention), those that did not involve any form of digital (e.g. computerized or app) or virtual reality delivery, those who did not assess clinical outcomes (e.g. imaging or EEG exclusively), those focusing on assessments of cognitive function without delivering treatment content, those comparing two digital rehabilitative strategies without a control group (e.g. to compare characteristics of digital interventions), and blended interventions (i.e. combined experimental intervention with any other form of intervention including face-to-face therapy (e.g. video, telemedicine)), unless the added intervention was provided in a standardized manner to both experimental and control groups (Table 4).

**Table 4:** Study eligibility criteria

Inclusion criteria	Exclusion criteria
▪ Randomised controlled trials ▪ Standalone digital interventions targeting cognitive symptoms ▪ Self-guided* ▪ Adult (≥18 years) populations with cognitive symptoms ▪ Compared with a control condition either inactive (treatment as usual or waitlist) or active (sham or traditional face-to-face therapy) ▪ Symptoms measured at post-treatment ▪ Minimum of 10 patients in each group ▪ Peer-reviewed	▪ Quasi-randomized studies or other than RCT ▪ Populations with a dementia diagnosis ▪ Major psychiatric disorders (e.g. schizophrenia) ▪ Not digital therapies ▪ Interventions requiring therapist guidance to deliver the treatment ▪ No cognitive outcomes assessed (e.g. imaging or EEG exclusively) ▪ Digital tools focusing on assessments of cognitive function without delivering treatment content ▪ Studies comparing two digital rehabilitative strategies without a control group ▪ Blended interventions (i.e. combined experimental intervention with any other form of intervention), unless the added intervention was provided in a standardised manner to both experimental and control groups

*Self-guided interventions require patients to independently engage with the intervention, while contact with therapists is only allowed at the start and/or completion of the intervention, and for sporadic telephone support, but not for the delivery of the intervention.

### Search strategy

Details on our search strategy can be found in Supplementary Table 5. Our comprehensive search strategy was developed by VC and LF, in consultation with a medical librarian. Key search terms included a combination of three major themes: cognitive disorders, digital/internet-delivered interventions and mobile health, and randomized controlled trials. We conducted the search in four different databases (EMBASE, Ovid Medline, PsycInfo and Cochrane Central Register of Controlled Trials), from inception to 2 June 2024. If a systematic review was identified in the search results the reference lists were searched for additional studies.

### Data extraction and synthesis

Duplicates were removed in Covidence. VC and TW screened the articles and extracted the data; disagreements were resolved through discussion with a third author (AC). Interrater reliability is reported for the title and abstract screening as well as full-text eligibility, where values of kappa are rated as fair (*κ* = 0.4–0.59), good (*κ* = 0.6–0.74), or excellent (*κ* > 0.75)^153^.

The following data was extracted into a spreadsheet format and then inputted into RevMan: authors, year of publication, country, study design (sample size, target population, type of control group, outcomes, treatment duration), sample characteristics (age, gender), treatment (theory framework and components, mode of delivery), and data for calculation of effect sizes (means and dispersion data, preferably intention-to-treat post-treatment data, of outcome data grouped into six categories: cognition, physical health/fatigue, performance of activities of daily living, mental health, quality of life outcomes, and study dropout). Disagreements were resolved through discussion.

### Quality assessment

Included articles were assessed by VC and TW using the revised Cochrane risk-of-bias 2.0 tool for randomized trials^154^: random sequence generation, allocation sequence concealment, blinding of participants and personnel, blinding of outcome assessors, incomplete outcome data, and selective reporting. Based on predefined definitions, studies with high risk of bias or some concerns in the different domains were considered as having a high risk of bias. Studies which were rated as low on all available criteria were rated as overall low risk of bias. Interrater reliability of the risk of bias assessment is reported.

### Statistical analyses

In the outcome analyses, we pooled studies with the same target outcome to generate a mean effect size for each outcome category. We selected either the primary outcome measure of each individual study or preferably a global scale rather than a subscale analyzing a particular domain. When possible, the same outcome measure or disorder-specific instruments were pooled across different trials. Both observer‐rated outcomes and patient‐reported outcomes were used (Supplementary Table 2).

For each comparison between a self-help digital intervention and a control condition, we calculated the effect size Hedges’ g (g), the 95% confidence interval (95%CI) and *p*-value (*p*) for each outcome type based on the post-assessment values or the difference between the pre- and post-assessment values (change-from-baseline). The effect of interest of a randomized trial was taken as the ‘intention-to-treat’ effect. A random-effects meta-analysis approach was used to examine the effect sizes given the expected heterogeneity across trials^155^. We combined studies for meta‐analyses, when at least two studies reported data for the same cognitive intervention framework (Table 1) and outcome category (Table 2), to allow for conclusions to be drawn. If trials were multi-armed, reporting two or more intervention groups to the same control comparison, we divided the sample size of the comparison to avoid inflating power. We adopted the formulae to estimate effects, and their standard errors, for the commonly used effect measures from Cochrane methodologies^126^: 95% confidence intervals for within-group comparisons were used to calculate standard deviations around the mean; mean difference was used for between-group comparison standard deviation calculation. We used odds ratio for dichotomous outcomes (dropout rate) and standardized mean difference (SMD) for continuous outcomes given that different measures were applied across studies. For g, negative values are interpreted as an improvement in function of the experimental group compared to the control group. When an outcome measure reflected improvement by scoring more (e.g. MoCA, EQ-5-D), the g values were inverted. SMD represents the effect size known as Hedges’ (adjusted) *g*: values of 0.2–0.5 are interpreted as small effect, 0.5–0.8 as moderate effect, and >0.8 as large effect^126^.

Statistical heterogeneity between of the effect sizes was assessed using the using forest plots and the I^2^ statistic^126^: low (25%), moderate (50%), or high (75%).

Indications of publication bias were evaluated by visual inspection of the funnel plot and by conducting the Egger’s test of asymmetry^156^.

Finally, potential sources of heterogeneity between trials/moderators of treatment effect were investigated by conducting subgroup analyses (control groups, population, delivery mode, treatment duration, and risk of bias). Study design and guidance were not included as moderators given the homogeneity of our inclusion criteria (RCT and self-guided interventions only). Six studies were not included in the meta-analyses, one because it was the single study in our review to focus on cognitive remediation^84^, and five because the authors did not report extractable data^62,86,101,109,122^.

All analyses were performed using Review Manager v5.4 and R 4.3.1.

## Supplementary information

Supplementary Information

Supplementary Data 1

Supplementary Data 2

## Data Availability

V.C. has full access to all of the data in the study and takes responsibility for the integrity of the data and the accuracy of the data analysis. The data used and analysed during the current study are available from the corresponding author on reasonable request.

## Code availability

Data extracted from included studies and data used for all analyses are provided in Supplementary Data files 1 and 2.
