# Supplementary material for: Systematic review and meta-analysis of standalone digital interventions for cognitive symptoms in people without dementia
Source: NPJ Digit Med. 2024 Oct 10;7:278. doi: 10.1038/s41746-024-01280-9 (PMC11467311; doi:10.1038/s41746-024-01280-9)
Supplement: Supplementary file 1 — Supplementary Information [file 41746_2024_1280_MOESM1_ESM.pdf]

Supplementary table 1. Characteristics of included studies.

Author, year	Population	Symptom duration	N (I/C)	Mean age	Female (%)	Intervention method	Duration	Number of sessions	Control(s)	Format	Outcomes
Hildebrandt, 2007	Multiple sclerosis	5 years	42 (17/25)	42	-	Cognitive training (VILAT-G 1.0, difficulty level individualized)	6 weeks	At least 5 days a week for 30 minutes/day	Usual care	Compact disc (CD)	WAIS, CVLT, PASAT, BDI, FSS, SF-36, SF-12
Barnes, 2009	MCI	-	47 (22/25)	74	40	Cognitive training (Posit Science)	6 weeks	100 minutes/day, 5 days/week	passive computer activities	Computerized	RBANS, CVLT-II, COWAT, BNT, TMT, Fluency tests, Spatial Span test, GDS
Mattioli, 2010	Multiple sclerosis	19 years	20 (10/10)	42	-	Cognitive rehabilitation (RehaCom package)	12 weeks	3 times per week, 1h/session	No intervention	Computerized	Spatial Recall Test, SDMT, PASAT, WCST, COWAT, MADRS; MSQoL
Optale, 2010	Older adults (>=65) with memory deficits, in a rest care facility	-	31 (15/16)	80	67	Virtual Reality memory training (Virtools platform)	24 weeks	3 auditory and 3 VR 30-min sessions every 2 weeks, over 3 months (36 sessions); followed by a booster	Face-to-face music therapy.	Virtual Reality/Computerized	MMSE, digit span, long term verbal memory, DTP, CET, TMT, CDT, ADL, GDS
Finn, 2011	Older adults with MCI (MMSE >=24)		25 (12/13)	74	64	Cognitive training (Lumosity)	11 weeks	30 sessions	Waitlist control	Computerized	CANTAB, MFQ, depression anxiety and stress scale
Åkerlund, 2013	Subacute acquired brain injury(stroke+ trauma) with working memory impairment	32 weeks (12-135)	45 (25/20)	52	49	Cognitive training (Cogmed QM)	5 weeks	-	Integrated rehabilitation only	Computerized	Digit span, Working Memory subscale; Barrow Neurological Institute Screen for Higher Cerebral

											Functions; HADS
Barnes, 2013	Inactive, community residing older adults with cognitive complaints (without dementia)	-	126 (63/63)	73	62.7	Cognitive training (intensive computer) plus exercise intervention (aerobic) or exercise control (factorial design)	12 weeks	1 h/day, 3 times/week	Educational DVDs	Computerized	Composite score (RAVLT, letter and category fluency, SDMT, TMT A/B, Eriksen Flanker Test, reaction times, Useful Field of View)
Gropper, 2014	ADHD or ADHD + learning disability	-	62 (39/23)	28	66	WM training (Cogmed)	5 weeks	25 training sessions (45 min/session)	Waitlist control	Compact disc (CD)	Digit Span, CANTAB, PASAT, Adult ADHD Self-Report Scale, CFQ
DeGiglio, 2015	Multiple sclerosis with cognitive complaints	13	35 (18/17)	44	74	Rehabilitation game (Nintendo console - http://www.nintendo.it/Giochi/Nintendo-DS/Brain-Training-del-Dr-Kawashima-Quanti-anni-ha-il-tuo-cervello)	8 weeks	30 min/d, 5 d/week	Waitlist group	Videogame	Stroop Test, PASAT, SDMT, MFIS, MSQoL-54
Gich, 2015	Multiple sclerosis with MCI	13y	43 (22/21)	45	71	Cognitive rehabilitation (MS-Line!)	24 weeks	two 75-minute sessions per week	No treatment	Computerized	SPART, SDMT, PASAT, WLG, FAS, WAIS-III, BNT, TMT; anxiety and depression; QoL
Mawjee, 2015	ADHD		97 (32/32)	24	60	Standard-length adaptive Cogmed Working memory training	5 weeks	45-min/session, 5 days/week	Waitlist-control group	Computerised	WAISIV Digit Span (auditory-verbal WM), CANTAB Spatial Span (visual-spatial WM) and WRAML Finger Windows (visual-spatial WM),

											Adult ADHD Self-Report Scale, CFQ
Moëll, 2015	ADHD and judged to currently have pronounced problems with organization and inattention	-	57 (29/28)	36	76%	Online course aiming at improving organizational skills and attention	6 weeks	7 modules distributed over 6 weeks	Waitlist control	App	ASRS, Sheehan disability scale (SDS), HADS, PSS, clinical significant change
Barban, 2016	MCI	-	348 but only 106 with MCI (46/60)	74	-	Cognitive training plus reminiscence therapy (RT) SOCIABLE (adjusted difficulty levels)	12 weeks	24 1-h sessions twice weekly	Rest (crossover design)	Computerized	RAVLT, RCF, TMT, PFT, MMSE, IADL
Gooding, 2016	Older adults with subclinical cognitive decline	-	96 (31/23/ 20)	76	42%	Cognitive training (Posit Science's BrainFitness)<br><br>And<br><br>Cognitive vitality training: computerised cognitive rehabilitation programme that is embedded within the Neuropsychological and Educational Approach to Remediation (NEAR) model of treatment	16 weeks	30 hours of training (60 minutes 2x/week)	Active control group (commercial ly available games and puzzles) (both intervention s were independent ly compared with ACG)	Computerised	MMSE, BSRT; memory tests, BDI-II
Hyer, 2016	Older Adults with MCI	-	68 (34/34)	75	53	Cognitive training (Cogmed QM)	5-7 weeks	25 sessions (~40 minutes/day)	Sham computer program	Computerized	Wechsler Memory Scale, TMT A/B, FAQ, CFQ
Lin, 2016	Older Adults with Amnestic Mild Cognitive Impairment	-	21 (10/11)	73	50	Cognitive training (INSIGHT online program (Posit Science))	6 weeks	1 hour/day, 4 days per week	Mental leisure activities: Online crossword, Sudoku, and solitaire games	Computerized	Useful Field of View, EXAMINER test, IADL
Wentink, 2016	Stroke patients with self-perceived cognitive impairment	26 months	110 (53/57)	59	36	Cognitive rehabilitation (Lumosity)	8 weeks	5 days/week, 15-20 minutes per day (total of 600 minutes)	Weekly information about the brain	Computerized	TMT-A/TMT-B, Block span task, digit span task, Eriksen Flanker Task, CFQ,

											SS-QoL-12, GSES
Bray, 2017	Cancer survivors with cognitive complaints	Adjuvant chemotherapy in the previous 6 to 60 months	243 (122/121)	53	95	Cognitive rehabilitation (INSIGHT)	15 weeks	40-minute sessions/week (total of 40 hours)	Standard medical care	Computerised	FACT-COG, PCI, Cogstate, anxiety and depression, QOL, fatigue and stress
Charvet, 2017	Multiple sclerosis with mild impairment on symbol digit modalities test	11.9	135 (74/61)	48	57	Adaptive cognitive remediation (ACR) (BrainHQ program)	12 weeks	1-h/day, five days/week (60 hours)	Active control condition (software gaming suite)	Computerized /tablet	Neuropsychological Composite Score, self-reported change in cognitive functioning
Hagovska, 2017	MCI	-	60 (30/30)	68	54	Cognitive training (CogniPlus)	10 weeks	Two 30-minute sessions per week (20 training sessions)	Classical group-based cognitive training	Computerized	FAQ, QOL, ACE, Stroop, MMSE
Han, 2017	MCI	-	50 (25/25)	74	47	Cognitive training (USMART) program (adjusted-difficulty)	4 weeks	30 min per session, twice per week, over the 4-week intervention period	Usual care (crossover trial)	App	Word List Memory Test, WLRT, and Word List Recognition Test; SMCQ; GDS; MMSE
Mawjee, 2017	ADHD	-	38 (18/8/12)	23	48%	Cognitive training (Cogmed)<br><br>Standard-length (used for analysis) and shortened-lenght	5 weeks	45 min of training, once a week	Waitlist control group	Computerised	Digit span, WAIS-IV, spatial span, CANTAB, Adult ASRS, CFQ, Barkley Deficits in Executive Functioning Scale–Short Form (BDEFS-SF)
Messinis, 2017	Multiple sclerosis with mild to moderate cognitive	13 years	58 (32/26)	46	69	Cognitive rehabilitation (RehaCom)	10 weeks	20 one-hour sessions (2 times/week)	Standard clinical care	Computerized	Composite cognitive scores

	impairment (MMSE<24)										
Mihuta, 2017	Cancer survivors with self-reported cognitive impairment	3 years since primary treatment	76 (40/36)	55	100	Cognitive rehabilitation (eReCog)	4 weeks	1 module per week (30-60 minutes)	Waitlist	Computerized	FACT-Cog, Prospective Memory, EORTC-QLQ-C30 FSS, BIPQ, Kessler Psychological Distress Scale
Petterson, 2017	ADHD	-	45 (13/18)	39	54%	Internet-delivered cognitive behavioral therapy (iCBT) in a self-help format (In Focus)	10 weeks	?	Wailist control	Computerized (internet-based)	Current Symptoms Scale—Self-Report Form, BDI-II, BAI, ADHD Impact Module–Adult (AIM-A), COPM
Savulich, 2017	Mild cognitive impairment in adults older than 45 years-old		42 (21/21)	75	17/42	Game Show Cognitive training	4 weeks	8 hours of gameplay	Usual care	Game	GDS, HADS. AES, CANTAB, MMSE, Brief Visuospatial memory test-revised
van de Ven, 2017	Stroke patients with cognitive impairment	3 months to 5 years after stroke	97 (38/35/ 24)	60	-	Cognitive flexibility training (Braingymmer)	12 weeks	58 (five times/week for half-hour) sessions (total of 29 hours)	Mock training or waitlist	Computerized	TMT, number-letter switching, Category fluency, letter fluency, Tower of London, Switch-task, PASAT, DSC, RAVLT
Arsoy, 2018	Multiple sclerosis	13 years	21 (10/11)	37	71	Cognitive rehabilitation (NOROSOFT Mental Exercise Program)	24 weeks	5 days/ week (50 min/each)	Usual care	Computerized	BRB-N, Stroop test, BDI, 9-hole peg and timed 25-foot walk tests

Oh, 2018	Older adults with subjective memory complaints and MMSE≥24		60 (18/16)	59	53	Cognitive training (SMART)	8 weeks	15-20 minutes per day, five days per week	Compared against the waitlist control arm	App	MMSE, Korean WAIS, SCWT, CES-D, STAI, MMQ
Pereira-Morales, 2018	Older adults with subjective memory complaints, MMSE ≥ 21		49 (17/15)	66	90	Cognitive training (http://app-cerebroactivo.rhcloud.com)	8 weeks	60 min/day, 4 days/ week	Treatment as usual (received information brief brochure only)	Computerized	SMCQ, MMSE, Grober and Buschke test, WAIS, digit span and digits and key symbols, CDT, TMT-B SCWT, verbal fluency test; GDS, STAI
Stuifbergen, 2018	Multiple sclerosis		183 (90/93)	50	87	Cognitive rehabilitation (Lumosity)	8 weeks	Computer training program (3 sessions (45-60 min of training) a day three times a week, plus group sessions (2 h per week for 8 weeks)	Usual care plus computer games (“MyBrainGames”)	Computerized	MACFIMS, PROMIS v1.0-Applied Cognition-Abilities, MMQ-Strategy, EPT-R, 17-item General Self-Efficacy Scale, CES-D
Vanderploeg, 2018	Mild TBI	9.5 months post-TBI	126	31	-	Plasticity-based computer cognitive rehabilitation (http://dvbic.dcoe.mil/research/studymanuals)	6 weeks	10 hours/week	(a) psychoeducation, (b) therapist-directed manualized CR, and (c) CR integrated with cognitive–behavioral therapy	Computerized	PASAT, SCL-90–R, GSI, KBCI
Wu, 2018	Prostate cancer patients on	7 years	60 (40/20)	67	-	Cognitive training (BrainHQ)	8 weeks	1 h/day, 5 days/week	Usual care	Computerized	PAOFI,

	androgen deprivation therapy										46-item Frontal Systems Behavior Scale, FrSBe
Bernini, 2019	Parkinson's disease (MCI)		41 (23/18)	71	56	Cognitive rehabilitation (CoRe)	4 weeks	3 sessions/week, each lasting 45 minutes	standard therapy	Computerized	MOCA, MMSE, BDI, PDQ-8
Bo, 2019	Stroke	< 6 months post-stroke	225 (57/57)	66	47	Cognitive rehabilitation (COGPACK neurorehabilitation programme, Marker Software)	12 weeks	60 minutes of training, 30 times weekly	Usual care plus video documentaries 45-minute session	Computerized	TMT-B, DST-F
Çinar, 2019	Subjective cognitive impairment	-	60 (30/30)	70	56	Cognitive training (BEYNEX)	1200 minutes	15-29 minutes/day	Standard medical care	Computerized	MoCA, Bayer-ADL, CANTAB, GDS
De Luca, 2019	Parkinson's disease	-	60 (30/30)	62	47	Cognitive rehabilitation (ERICA Platform)	8 weeks	3 sessions a week, 60 minutes/session	Face-to-face interaction and paper and pencil activities	Computerized	ACE-R, FAB; WEIGL; HAMA; GDS
Darestani, 2019	Multiple sclerosis	-	60 (30/30)	38	72	Cognitive rehabilitation (RehaCom software, autoadaptive difficulty level)	5 weeks	10 (2 sessions per week, 1 h each)	Control group	Computerized	COWAT, CVLT-II
Li, 2019	MCI	-	160 (80/80)	70	47	Cognitive training	24 weeks	3-4 times/week (120–160 min training in total)	Control	Computerised	MMSE, ACER, AVLT, shape trail test (Part A and B), CFT, SDMT, SCWT
Song, 2019	Cognitive dysfunction after lung transplant in patients older than 55 years-old (MoCA >26 points or MMSE ≥24 points)	Fifth week after the surgery	46 (23/23)	66	30	Cognitive training (Posit Science BrainHQ suite)	8 weeks	4 tasks/day, 10 minutes per task, 5 days per week	Control	Computerized	Digit-Span Forward and Backward Test, Verbal Fluency Test, TMT-A/B, Digit Symbol Test, Word Recognition Test

Yang, 2019	Older adults with MCI (MoCA ≥23)	-	66 (33/33)	79	79	Cognitive training (CogniPlus)	12 weeks	45 min sessions, 3 times/week	active control group (reading online e-books and playing online Games)	Computerized	working memory; Wechsler, MMQ, MMSE, MoCA
Bellens, 2020	Breast cancer survivors with cognitive impairment	-	46 (23/23)	51.8	-	Videogame (Aquasnap, Cambridge, MyCQ™)	12 weeks	three times a week for a minimum of 60 min of game playing per week	Waitlist control receiving supportive care (crossover trial)	Videogame	MyCQ™ cognitive score, ADL, HADS, BCIS, CFQ, PSQI, RAND36
Chmelarova, 2020	Multiple sclerosis	-	43 (26/17)	41	79	Cognitive rehabilitation (HAPPY neuron Brain Jogging)	8 weeks	4 times per week/30 min per session	Standard medical care	Computerized	RBANS, CFQ, Schwartz SOS 10 scale, BDI, HAMD
Fellman, 2020	Parkinson's disease patients (without dementia)	5.6 years	54 (26/28)	65	65	Cognitive training	5 weeks	30-min sessions, 3 times per week	Active control (free online quiz training Älypää; "classic" game mode)	Computerised	Working Memory Questionnaire, BRIEF-A, GDS-30
Liao, 2020	Older adults (≥65 yo) with MCI	-	34 (18/16)	75	68	Virtual reality (VIVE and Kinect systems, USA)	12 weeks	3 sessions/week, 60 minutes/session	Combined physical and cognitive training	Virtual reality	MoCA, EXIT-25, CVVLT, Lawton Instrumental Activities of Daily Living scale
Messinis, 2020	Multiple sclerosis with cognitive deficit on at least two domains of the Central Nervous System Vital Signs	21	36 (19/17)	47	63	Cognitive rehabilitation (RehaCom software)	8 weeks	3 times per week, 45 min/session	Active control group (sham computer based activities plus usual care)	Computerised	BICAMS, BDI-FS, MFIS, EuroQol EQ-5D VAS
Park, 2020	Amnestic MCI and age between 50 and 80 years	-	21 (10/11)	72	73	Virtual reality (HTC Vive, New Taipei City, Taiwan)	12 weeks	30 min/day, two days/week (total 24 sessions)	Waitlist control	Virtual reality	K-MMSE, GDS, digit span, stroop test, fluency tests, Seoul

											Neuropsychological Screening Battery, Dementia version
Thapa, 2020	MCI >55 years-old	-	68 (34/34)	73	76	Immersive virtual reality cognitive training (SY Innotech Inc., Busan, South Korea)	8 weeks	Three 20 min sessions, 3 times/week (total duration 100 minutes or 24 sessions)	Educational program on general health care once a week	Virtual reality	MMSE, TMT-A/B, SDMT, Gerontology Functional Assessment tool, gait speed and 8-feet Up and Go
Vilou, 2020	Multiple sclerosis	8	47 (23/24)	34	87	Cognitive rehabilitation (BrainHQ)	6 weeks	Two 40-minute sessions per week	Usual care	Computerized	BICAMS, SCWT, TMT A/B
Yang, 2020	Older adults with MCI	-	78 (39/39)	80	-	Cognitive training (CogniPlus)	6 weeks	18 sessions (45 minutes/session, three sessions/week)	Active control group (tablet computer with cognitive games (spot the difference, jigsaw puzzle, and memory card game)	Computerized	Trail making test A and B, reaction time, domains of MoCA and MMSE
Blair, 2021	Multiple sclerosis	14.9 years	30 (15/15)	51	70	Cognitive training (Cogmed)	5 weeks	25 training sessions daily, 30-45 minutes/session	Standard medical care	Computerised	PASAT, SDMT, DKEFS Color-Word Interference Test and other cognitive measures (secondary), CFQ, SF-36, Fatigue Severity Scale, BDI, HADS, numeric pain scale

Bernini, 2021	Parkinson's disease (MCI)	8.44 years	53 (21/18)	74	33	Cognitive rehabilitation (CoRe software)	3 weeks	4 sessions/week	Active control groups - traditional paper-and-pencil cognitive training (PCT), or an unstructured activity intervention	Computerized	MMSE, MoCA, Logical Memory Test, immediate and delayed recall, RCF, SPM, FAB, semantic and phonological fluency, working memory tests, Attentive Matrices, TMT A/B
De Luca, 2021	Multiple sclerosis (MCI)	8	40 (20/20)	53	40	Cognitive rehabilitation (ERICA)	8 weeks	3/week (45 minutes each)	Active control group - Traditional cognitive rehabilitation	Computerized	MoCA, DSMT, PASAT, STRT-CLTR Selective Reminding Test - Consistent Long Term Retrieval; SRT-D Selective Reminding Test - Delayed Recall of the Selective Reminding Test; SRT-LTS Selective Reminding Test - Long Term Storage, SPATIAL recall test, WLG, BDI, HRS-A, MSQOL
Kang, 2021	Subjective cognitive decline or	-	41 (23/18)	75	71	Virtual reality (fully immersive VR cognitive training)	4 weeks	2/week (20-30 min/each)	Usual care	Virtual reality	RCF; BNT, TMT A/B, MMSE,

	mild cognitive impairment (>60 yo)										SVLT, SCWT, GDS, AES, PANAS, QoL-AD, tolerability
Kim, 2021	Acute or subacute traumatic brain injury with executive dysfunction	25.1±18days	32 (18/17)	66	50	cognitive rehabilitation (RehaCom)	2 weeks	30 minutes/day, 5 times/week	therapist-driven cognitive rehabilitation (TCR)	Computerized	Digit symbol coding and searching tests, TMT-A/B, COWAT, phonetic and semantic verbal fluency, MMSE, MoCA, MBI
Leonardi, 2021	Multiple sclerosis patients with mild to moderate cognitive impairment		30 (15/15)	57	47	Virtual reality (VRRS-Evo)	8 weeks	3 times a week (24 sesions, 45 min/each)	Conventional cognitive rehabilitation with a therapist	Virtual reality	BDI, HAM-A MOCA, BRB-N, MSQoL-54
Mahncke, 2021	Mild traumatic brain injury and cognitive impairment	7 years post-injury	83 (41/42)	34	19	Cognitive training (BrainHQ)	13 weeks	5 days per week, 1h/day	Active control (computer games)	Computerized	composite of nine standardized neuropsychological assessments, TIADL; SF-12, BDI, Post-Traumatic Stress Disorder Checklist, Frontal Symptoms Behavioral Scale, CFQ, NPI
Peers, 2021	Stroke patients	38 months	80 (27/27)	58	-	Cognitive training (attention training and working memory training groups)	4 weeks	20 sessions of 20 minutes/day	Waitlist	Computerized	TVA (spatial bias), visual short-term memory capacity, OCS-BRIDGE (cognition

											and mood), EBIQ, CFQ, Subjective neglect questionnaire
Tarantino, 2021	Stroke	3 months since the event	37 (18/19)	65	30	Cognitive rehabilitation (inspired by the ROBBIA brain-based model)	15 days	10 sessions, 1 hour/each	Usual rehabilitation only	Computerized	Digit span forward and backward, Corsi block-tapping test; Attentional matrices, TMT-A/B; BNT; Phonemic and Semantic fluency, WCST, Five Point test, SCWT; MBI; Functional Independence Measure
Torpil, 2021	Older Adults with MCI		64 (32/32)	70	59	Virtual reality (VR) - based rehabilitation program in addition to a conventional cognitive rehabilitation intervention (Microsoft Kinect for PC program without immersion)	12 weeks	45-minute sessions twice a week	Face-to-face cognitive rehabilitation	Virtual reality/computerized	Loewenstein Occupational Therapy Cognitive Assessment -Geriatric
Van der linden, 2021	Low-grade glioma or meningioma	three months after resective surgery	62 (31/31)	46	74	Cognitive rehabilitation (ReMind-app)	10 weeks	three hours a week	Waitlist group	App	Digit span test, Wechsler, letter fluency; CFQ, BRIEF-A, MFI-20, HADS
Ho, 2022	Stroke and mild to moderate cognitive impairment	20 months after stroke	39 (19/20)	63	33	Cognitive training (Lumosity)	12 weeks	20 min, twice a week (total of 24 sessions)	paper-and-pencil tasks and tabletop tasks	Computerized	MMSE, SDMT, Digit Span Test, spatial span test, MoCA, Stroke impact scale (QoL and ADL)
Kozora, 2022	Systemic lupus	13 years	60 (30/30)	39	93	Videogame–based interface (EVO Monitor)	4 weeks	25 minutes, 5 times a week	Treatment as usual	Tablet-based videogame	Wechsler Test of Adult

	erythematosus										Reading, WAIS-IV, TMT-A, TMT-B, Stropp color and word test, digit vigilance test, response time, VAS (0-10) scores for depression, anxiety, pain, and fatigue
Liu, 2022	Post-Stroke Cognitive Impairment	43 months	30 (15/15)	75	43	Immersive virtual reality-based puzzle game	6 weeks	6 times a week, 15 minutes per session	Active control group (traditional cognitive training)	Virtual reality	MoCA, TMT-A, DSST, DST, VFT, Barthel index
Park, 2022	Amnestic MCI	-	56 (28/28)	72	50	Virtual Reality	8 weeks	24 sessions (45 minutes a session, 3 days a week)	Waitlist control group	Virtual reality in a computer	WAIS, SVLT
Pottgen, 2022	Multiple sclerosis	9 years	176 (68/108)	42	66	Cognitive training (BrainStim) in combination with metacognitive training (group)	4 weeks	2 times a week (45-60 minutes/session)	Active control (Standard rehabilitation plus metacognitive training (group))	Computerized	Perceived Deficit Questionnaire (PDQ), BICAMS, WMS, HADS, Fatigue Scale for Motor and Cognitive Functions (FSMC), coping self-efficacy scale, Hamburg Quality of Life Questionnaire in Multiple Sclerosis (HAQUAMS), and Frenchay

											Activity Index (FAI)
Von Ah, 2022	Breast cancer survivors	64 months since diagnosis	46 (22/24)	58	-	Cognitive training (BrainHQ)	10 weeks	40h of training	attention control program: crossword puzzles	Computerized	Cognitive Abilities and Cognitive Concerns 8-item, SF-36, RAVLT, Rivermead Behavioral Paragraph Recall Test, Digit Span, WAIS, SDMT, COWAT
Baldimtsi, 2023	Mild cognitive impairment	-	56 (28/28)	71	81	Virtual reality (VRADA)	12 weeks	2-3/week (20-30 minutes, maximum 32 sessions)	No contact control group	Virtual reality	MMSE, RAVLT, WAIS, digit span, TMT-B
Feinstein, 2023	Multiple sclerosis	14 years	154 (79/75)	52	62	Cognitive rehabilitation (RehaCom) plus sham exercise (stretching)	12 weeks	2 times a week (mean of 41 minutes)	Sham cognitive rehabilitation (internet searches) plus sham exercise (stretching)	Computerized	SDMT, CVLT-II, Brief visuospatial memory test, 6MWT, HADS, fatigue impact scale, EuroQol EQ5-VAS, 20-item perceived deficits questionnaire, 12-item MS walking scale, 29-item MS impact scale, global functional (functional assessment of MS)
Galperin, 2023	Multiple sclerosis	-	124 (64/60)	49	72	Virtual reality + treadmill	6 weeks	3 times a week (maximum 13-18)	treadmill training alone (TT) (active-	Virtual reality	SDMT, dual task gait speed, 25-Foot Walk

									control) group		(T25FW), 6MWT, BICAMS, CVLT-II BVMTR, WLG, TMT-A, TMT-B, Multiple Sclerosis Walking Scale-12 (MSWS-12), MSQOL, PHQ-9
Goumopoulos, 2023	Mild cognitive impairment	-	21 (11/10)	73	52	Virtual reality	12 weeks	2 times/week (60 minutes, 24 sessions)	Treatment as usual	Virtual reality	MoCA, RAVLT, TMT-A/B, DST, CDT, FAQ, IADL, GDS
Kim, 2023	Middle-Aged Women with subjective cognitive symptoms/MCI	-	60 (30/30)	55	100	Virtual reality	12 weeks	2 times/week (30 minutes, 24 sessions)	Active control group (cognitive training regular activities)	Virtual reality	MoCA, DST, Stroop test, CWST, GDS
Maeir, 2023	Cancer survivors with cognitive impairment	34 months	49 (25/24)	51	75	Cognitive training arm (BrainHQ)	12 weeks	3 times/week (25 minutes/session)	Treatment as usual	Computerized	COPM, FACTcog, FACT-GP
Nousia, 2023	Amnestic MCI	-	36 (19/17)	75	50	Cognitive rehabilitation (RehaCom)	15 weeks	2 times/week (60 minutes each)	Treatment as usual	Computerized	MoCA, digit span, BNT, semantic fluency, TMT-A, TMT-B, CDT
Baik, 2024	Mild cognitive impairment	-	50 (25/25)	67	68	Cognitive training (Neuro-World)	8 weeks	3 times/week (maximum 24 sessions, 24 minutes each)	Treatment as usual	Computerized	MoCA, verbal learning tests, DST, semantic fluecy, phonemic fluency, GDS
Klaver, 2024	Cancer survivors	3 years since diagnosis	186 (93/93)	49	83	Cognitive rehabilitation (“Niet-Rennen- Maar-Plannen”)	12 weeks	-	Waitlist-control	Computerized	Work Ability Index, WRFQ, Cognitive Symptom Checklist-

											Work Dutch Version, VBBA, SF-36 General perception

Addenbrooke’s cognitive examination (ACE), activities of daily living (ADL), ADHD Impact Module–Adult (AIM-A), ADHD self-assessment scale (ASRS), Beck Anxiety Inventory (BAI), Barkley Deficits in Executive Functioning Scale–Short Form (BDEFS-SF), Beck Depression Inventory (BDI), Brief International Cognitive Assessment for MS (BICAMS), Boston naming test (BNT), BRIDGE (cognition and mood), Buschke Selective Reminding Test (BSRT), Short Repeatable Battery of Neuropsychological Test (BRB-N), Brief Repeatable Battery of Neuropsychological Tests (BRB-N), Behavior Rating Inventory of Executive Function for Adults (BRIEF-A), Cambridge Neuropsychological Test Automated Battery (CANTAB), Clock-Drawing Test (CDT), Center for Epidemiological Studies-Depression (CES-D), Cognitive Estimation Test (CET), The Cognitive Failures Questionnaire (CFQ), Canadian Occupational Performance Measure (COPM), Controlled Oral Word Association Test (COWAT), California Verbal Learning Test (CVLT-II), Composed of digit forward (DST-F), EORTC Quality Of Life Questionnaire (QLQ-C30), European Brain Injury Questionnaire (EBIQ), Everyday problems test (EPT), Executive Interview (EXIT25), Frontal Systems Behavior Scale (FAB), functional assessment of cancer therapy-cognitive (FACT-Cog), F-A-S phonetic fluency test, Frontal Systems Behavior Scale (FrSBe), Fatigue symptom scale (FSS), Geriatric Depression Scale (GDS), Rasch analysis of the General Self-Efficacy Scale (GSES), Global Severity Index (GSI), Hospital Anxiety Depression Scale (HADS), Hamilton Anxiety Rating Scale (HAM-A), Brief Illness Perception Questionnaire (IPQ), Key Behaviors Change Inventory (KBCI), Montgomery–Åsberg Depression Rating Scale (MADRS), minimal neuropsychological assessment of MS (MACFIMS), Mild behavioral impairment (MBI), Modified Fatigue Impact Scale (MFIS), Memory failures questionnaire (MFQ), Metamemory questionnaire (MMQ), Mini Mental Status Examination (MMSE), MSQoL, Neuropsychiatric Inventory (NPI), Positive and Negative Affect Schedule (PANAS), Parkinson’s Disease questionnaire (PDQ-8), Paced Auditory Serial Addition Test (PASAT), Patients Assessment of Own Functioning Inventory (PAOFI), Pittsburgh Sleep Quality Index (PSQI), RAND 36-Item Health Survey (RAND-36), Rey Auditory Verbal Learning Test (RAVLT), Rey Complex Figure (RCF), Repeatable Assessment of Neuropsychological Status (RBANS), Rivermead behavioral memory test (RBMT), Sheehan disability scale (SDS), Short form survey (SF-36, SF-12), Subjective memory complaints questionnaire (SMCQ),Spatial Recall Test (SPART), Stroop Color and Word Test (SCWT), Symbol digit (SDMT), Symptom Checklist-90 Revised (SCL-90–R), STRT-CLTR Selective Reminding Test - Consistent Long Term Retrieval; SRT-D Selective Reminding Test - Delayed Recall of the Selective Reminding Test; SRT-LTS Selective Reminding Test - Long Term; Storage Schwartz Outcome Scale-10 (SOS-10), State-Trait Anxiety Inventory (STAI), Seoul Verbal Learning Test (SVLT), trail making test (TMT A/B), theory of visual assessment test (TVA - spatial bias), Wechsler intelligence scale (WAIS), Wisconsin Card Sorting test (WCST), Weigl Colour-Form Sorting Test (WEIGL), word list generation (WLG), Wide range assessment of memory and learning (WRAML), Work Role Functioning Questionnaire (WRFQ), Experience and assessment of work questionnaire (VBBA)

Supplementary Table 2. Difference between groups (inter-group analysis) at post-treatment, per individual outcome measured in the studies. (+) indicates a statistically significant benefit obtained in the experimental group; (-) indicates a statistically significant detrimental effect observed in the experimental group; (ns) denote non-significant changes between groups. Outcome measures extracted for meta-analysis are listed first in each category. Studies marked with * were not included in the meta-analysis due to incomplete data for calculation.

Study	Trained domains	Cognition	Physical function/ fatigue	Activities of daily living	Mental health	Quality of life	Treatment drop-outs
Hildebrandt, 2007	Memory and working memory	PASAT (+) Learning trials (CVLT) (+) Long delay free recall (CVLT) (+)	Fatigue (ns) Nine Hole Peg Test (MSFC) (+)	-	BDI (Ns)	SF-12 bodily score (Ns)	-
Barnes, 2009	-	RBANS total score (ns) Spatial span (+)	-	-	Not reported	-	5/22 intervention 6/25 controls
Mattioli, 2010	Divided Attention	PASAT 2'' (+) WCST (+) Cowa (+) Tea (+)	-	-	MADRS (+)	MSQoL (ns)	-
Optale, 2010	Memory (auditory stimulation) and visuospatial function	MMSE (+) Mental status in Neurology (+) Digit Span (+) VSR (+), PVF (+), DTP (+), CET (+)	-	Instrumental activities of daily living (ns)	GDS (+)	-	0/15 1/16
Finn, 2011	Attention, processing speed, visual, memory	CANTAB - Visual sustained attention (RVP A) (+) MFQ (n.s.) MCI (n.s.)	-	-	DASS21 (n.s.)	-	4/12 5/13
Åkerlund, 2013	Working memory	BNIS (+) Digit span (+) Working memory (+) Executive function (ns)	-	-	HADS D (ns)	-	3/25 2/20
Barnes, 2013 (comparison without exercise)	-	Reaction time (ns) RAVLT (ns) Verbal fluency (ns) Digit symbol test (ns) Processing speed (ns) TMT A/B (ns) Visuospatial function (UFOV) selective attention tasks (+), divided attention and processing speed (ns) Composite cognition score (ns)	-	-	-	-	6/31 8/32
Gropper, 2014	Auditory-verbal and visual-spatial working memory (adaptative)	WAIS-IV Digit Span (+) CANTAB spatial span (+), other domains of CANTAB (ns) PASAT (ns) CFQ (+)	-	-	-	-	5/39 0/23

		Adult ADHD Self-Report Scale (+)					
DeGiglio, 2015	-	Stroop test (+)	Cg-MFIS (ns)<br>Ps-MFIS (ns)<br>Ph-MFIS (ns)	-	Mental health composite score (+) (patients with depression and anxiety were excluded)	MSQoL-54 (ns) except emotional wellbeing (+) health distress (+) subscales	0/18<br>1/17
Gich, 2015	-	10/36 SPART-T (+)<br>10/36 SPART-DR (+)<br>WLG test (+)<br>LNS (+)<br>BNT (+)<br>TMT-A (+)<br>Digit span (ns)	-	MSIS-29 (ns)	HADS (+)	VAS of EuroQol-5D (ns)	1/22<br>1/21
Mawjee, 2015 (waitlist control)	Working memory	Digit span (+)<br>CANTAB (+)<br>ADRS (ns)<br>CFQ (ns)<br>Other cognitive measures (ns)	-	-	-	-	7/32<br>3/32
Moëll, 2015 (waitlist control)	Organisation skills, G-tasks, N-back tasks to train WM and fluid intelligence, simple noise to improve concentration	ASRS-Inattention and Hyperactivity (+)	-	Sheehan Disability Scale (ns)	HADS depression (+)<br>HADS anxiety (ns)<br>PSS (ns)	-	3/29<br>1/28
Barban, 2016	-	MMSE (ns)<br>Rey words delayed recall (+)	-	IADL (ns)	-	-	No data for MCI only
Gooding, 2016 (cognitive training)	Repeated drill-and-practice exercises involving memory, attention, and executive functions (adaptative)	MMSE (+)<br>verbal memory and learning (+)	-	-	BDI (ns)	-	-
Gooding, 2016 (cognitive rehabilitation)		MMSE (+)<br>verbal memory and learning (+)	-	-	BDI (+)	-	-
Hyer, 2016	Working memory	Span Board (non-verbal working memory) (+)<br>Cognitive Failures Questionnaire (CFQ) (+)	-	Functional Activities Questionnaire (FAQ) (+)	-	-	5/34<br>4/34
Lin, 2016	Vision-Based Speed-of-Processing Training	Mean reaction time (+)<br>Working memory (+)	-	Timed IADL (+)	-	-	-
Wentink, 2016	Attention, speed, memory, flexibility and problem solving	Working memory (Block span test) (+)<br>Reaction time incongruent) (+)<br>TMT A/B (ns)<br>Flexibility and attention (ns)<br>Self-efficacy measures (ns)<br>CFQ (ns)	-	-	-	SSQoL (ns)	3/53<br>0/57

Bray, 2017	Exercises targeting processing systems aimed at improving cognition through speed and accuracy of information processing)	FACT-COG perceived cognitive impairment (+) Comments From Others on Cognition (+) Objective assessment of cognitive domains or total score (Cogstate) (ns)	Fatigue FACT-F (+)	-	Anxiety and depression GHQ (+) Stress PSS (+)	Global QOL (ns) Impact on QOL from perceived cognitive impairmen t (+)	27/122 23/121
Charvet, 2017*	Speed, attention, working memory, and executive function through the visual and auditory domains	Cognitive functioning composite score (+)	-	-	-	-	4/74 1/61
Hagovska, 2017	Attention, working memory, long-term memory, planning of everyday activities, and visual-motor abilities	ACE score (+) Language domain (+) Stroop test (errors) (+) No group differences on the other subscales (i.e., memory, attention and concentration, verbal fluency, visuospatial abilities)	-	Functional Activities Questionnaire (n.s.)	-	Spitzer QOL (+)	0/30 2/30
Han, 2017	Memory (Spaced Retrieval)	Word List Recall Test (+) Other cognitive measures or MMSE (ns) SMCQ (ns)	-	-	GDS (ns)	-	2/25 5/25
Mawjee, 2017	Auditory-verbal and visual-spatial working memory	WAIS-IV (ns) CANTAB (ns) No significant differences in any of the other cognitive measures	-	-	-	-	10/18 3/12
Messinis, 2017	-	Composite cognitive domain scores, including SRTLTS, SRTDR, SDMT, BVMT-R, VFT, TMT-A, TMT-B and SNST-colour word task (+)	-	-	-	-	No dropouts
Mihuta, 2017	Psychoeducati on, relaxation, strategy training	FACT-COG perceived cognitive impairment (ns) Executive function and other cognitive domains (ns)	Fatigue symptom scale (ns)	IADL subscale assessing prospective memory failures (+) BADL (ns)	Distress (ns) BIPQ (ns)	QoL (ns)	8/40 3/36
Petterson, 2017	Compensatory techniques, such as gauging attention span,	Current symptom scale (+)	-	COPM (ns)	BDI (ns) BAI (ns)	ADHD Impact Module–Adult (AIM-A) (ns)	2/13 3/18

	behavior analyses, time management, problem solving, organization and planning, plus dysfunctional thinking (cognitive restructuring) and emotional distress (mindfulness and acceptance techniques)						
Savulich, 2017*	Memory and learning	Cambridge Neuropsychological Test Automated Battery Paired Associates Learning first trial memory score and errors (+), Mini-Mental State Examination (+), the Brief Visuospatial Memory Test (+)	-	-	Apathy Evaluation Scale (+) GDS (ns)	-	No dropouts
van de Ven, 2017	Working memory, attention, and reasoning (nine tasks in the cognitive domains of working memory, attention, and reasoning)	Overall cognition (ns) Trail making test (ns) Other cognitive measures including semantic fluency (ns)	-	-	-	-	8/38 5/35 4/24
Arsoy, 2018*	Attention, memory, reasoning, visual, and verbal tasks	SDMT (+) COWAT (+) Stroop test (+) PASAT-3 score (ns)	9 hole peg test (n.s.)	-	BDI (ns)	-	-
Oh, 2018 (waitlist control group)	Memory	Working memory quotient (+) Auditory-verbal WM score (+) TMT-A and TMT-B (ns) Executive Function Quotient (ns)	-	-	CES-D (ns) STAI-S (ns)	-	1/18 4/16
Pereira-Morales, 2018	Orientation, attention, short- and long-term memory, episodic memory and executive functioning	Grober and Buschke Short-term memory test (+) Other cognitive measures improved with the alternative active intervention only	-	-	STAI (+) GDS (ns)	-	5/17 4/15
Stuifbergen, 2018	-	3-sec PASAT (+) CVLT Delayed score (+) PROMIS Cognitive Abilities (+)	-	-	CESD (+)	-	12/90 8/93

Vanderploeg, 2018* (against active control group psychoeducation or CBT)	-	Self-administered computer cognitive rehabilitation was not only not beneficial, but negatively associated with cognitive and neurobehavioral improvement: PASAT (-)			KBCI (-)		
Wu, 2018	Visual attention and information processing exercises	Reaction time (+) cognitive flexibility and self-reported cognitive functioning (n.s)<br><br>Supression of memory temporarily that then returns to normal in the intervention group (-)	-	-	Frontal Systems Behavior Scale (n.s.)	FACT-P, Functional Assessment of Cancer Therapy-Prostate (n.s.)	9/40<br>0/20
Bernini, 2019	Executive tasks plus physical rehabilitation	MoCA, Rey's 15-word test immediate and delayed recall, Weigl's Test, FAB, TMTA and Stroop Test time interference and error interference (+)<br>No difference in other cognitive measures.	-	-	BDI (ns)	PDQ-8 (ns)	6/23<br>0/18
Bo, 2019	Visual-motor, learning, memory, attention and executive function	Forward digit span (+)	-	-	-	-	24/57<br>20/57
Çinar, 2019	3 different 5-min-long computer games,<br>a 3-min-long physical exercise video	MoCA (ns), CANTAB (+)	-	Bayer-ADL (ns)	GDS (ns)	-	-
DeLuca 2019	-	ACE (+)<br>WEIGL (+)<br>FAB (ns)	-	GDS (ns)<br>HRS-A (ns)	-	-	-
Darestani, 2019	-	CVLT-II (+)<br>COWAT (+)	-	-	-	-	-
Li, 2019	Visual working memory, episodic memory, speed of calculation, visual search, mental rotation, alertness	MMSE (+), ACE (ns), Complex figure test copy (+), Stroop test (ns), Shape trial (ns), symbol digit (ns)	-	-	-	-	2/80<br>17/80
Song, 2019	Attention and information processing speed, plus working memory	Digit-Span Forward Test (ns)<br>Verbal Fluency Test (ns)	-	-	-	-	2/23<br>0/23
Yang, 2019	Working memory	Digit Span backward (+)	-	-	-	-	4/32<br>2/32

		MMSE (+) MMQ Strategy (+) No difference in other cognitive measures					
Bellens, 2020	-	CFQ (self-reported cognitive failure) (+) reaction time, visual memory recognition, N back 1 and 2, coding, trail making test B, MyCQ score (correlated with CANTAB) (ns)	-	Limitations in physical and social functioning (ns)	Anxiety and depression (ns)	Pain (RAND36/ SF-36) (ns) PSQI (ns)	16/38 who completed the baseline assessment overall
Chmelařová, 2020	Memory, concentration, speech, logical thinking, spatial orientation	RBANS 2 total (+) TMT A/B (ns) CFQ (ns)	-	-	BDI (ns) Somatic and mental well-being (ns) HAMD (ns)	-	-
Fellman, 2020	Working memory: n-back training task, selective Updating of Sentences Training, and a Forward Simple Span Training	WMQ (ns) Trained working memory tasks (+) Task-specific near transfer composite score (+)	-	-	GDS-30 (+) BRIEF-A (ns)	-	-
Liao, 2020	VR-based exercise and cognitive training	MoCA (ns) EXIT-25 (ns) CVVLT (ns) Immediate and Delayed recall (ns)	-	IADL (+)	-	-	3/21 5/21
Messinis, 2020	-	Symbol Digit Modalities Test (+) GVLT = Greek Verbal Learning Test (+) BVMT = Brief Visuospatial Memory Test – Revised (+)	MFIS = Modified Fatigue Impact Scale Total and subscales (+)	-	BDI (+)	EQ5-VAS (+)	-
Park, 2020	Attention, processing speed, executive function and memory	MMSE (ns) Digit span (ns) Stroop test (ns) Fluency tests (ns)	-	-	GDS (ns)	-	-
Thapa, 2020	-	MMSE (ns) TMT-A (ns) TMT B (+) SDST (+)	Gait speed (+) 8-feet Up and Go (+)	-	-	-	1/34 1/34
Vilou, 2020	-	SDMT (ns) Greek Verbal Learning Test (+), TMT-A (+), Stroop (+) Brief Visuospatial Memory Test-Revised (+) TMT-B (ns)	-	-	-	-	-
Yang, 2020	Multidomain attention training: three attention modules (alertness, sustained attention, and visual-spatial attention	TMT-A/B (ns) Mean reaction time (ns)	-	-	-	-	0/39 0/39

Blair, 2021	Working Memory	PASAT (ns) SDMT (ns) DKEFS Color-Word Interference Test (ns) Letter Number Sequencing and Digit Span (ns)	-	-	HADS-D (ns) HADS-A (ns) BDI (ns)	SF-36 (ns)	4/15 2/15
Bernini, 2021	-	MoCA (+) MMSE (+) Attention, executive function (+) Working memory (ns) Episodic long-term memory (ns)	-	-	-	-	3/21 0/18
De Luca, 2021*	Attention process, memory abilities, spatial cognition, verbal and nonverbal executive functions	MoCA (+), SRT-LTS (+), SRT-CLTR (ns), SPART (ns), SDMT (+), PASAT (ns), SRT-D (+), WLG (ns)	-	-	BDI (ns)	MSQOL (ns)	-
Kang, 2021	Attention, executive function and memory, calculations, visuospatial functions, verbal and visual memory, processing speed and working memory	MMSE (ns) Stroop test (ns) RCFT (+) Naming (ns) COWAT semantic fluency (ns) COWAT phonemic fluency (ns) Immediate and delayed recall (ns) Digit span (ns) TMT A (ns) TMT-B (ns)	-	-	GDS (ns) AES (+) PANAS (+)	QoL-AD (+)	2/25 2/20
Kim, 2021	-	Phonetic fluency test (+) Symbol search (ns) MMSE (ns) MoCA (ns) TMT A/B (ns)	-	MBI (ns)	-	-	0/18 3/17
Leonardi, 2021	Memory, attention, Language, visuo-spatial orientation, calculation and executive function training – 2D and 3D exercises	MoCA (+) learning ability and verbal short-term memory (SRT-LTS) (+) lexical-access ability (WLG) (+) PASAT (ns) Symbol Digit Modalities Test (ns)	-	--	BDI (ns)	Quality of life- mental states (MSQOL MT) (+) Quality of Life-Physical (MSQOL-PH) (ns)	-
Mahncke, 2021	Speed/accuracy of information processing	Composite cognitive measure (+) CFQ (ns)	-	TIADL (ns)	BDI-II (ns)	SF-12 PCS/MCS (ns)	10/41 8/42
Peers, 2021*	Working memory or attention training	No differences in spatial bias or other cognitive measures	-	Function (+) (attention training group)	-	EBIQ (ns)	9/27 3/26 1/27
Tarantino, 2021	Executive function training in addition to the ordinary rehabilitation	Digit span (+) Phonemic fluency (+) errors on the WCST (+)	-	Barthel (+)	-	-	-

	program: working Memory, Interference Control and Inhibition, Task-Switching, and Monitoring	Stroop interference (IES) (+)						
Torpil, 2021	Orientation, attention/concentration, visual-spatial perception, reaction time, visuomotor organization, visual and spatial perception, cognitive time management, reaction time	Attention/concentration (+) Orientation (+) visual-spatial perception (+) visuomotor organization (+) thinking operation (+) Praxis (ns) Memory (ns)	-	-	-	-	2/32 1/32
vanderLinden, 2021*	Six modules: (1) Cognitive functions (2) Influences (3) Compensation , (4) Attention, (5) Planning & Control, (6) Memory	No inter-group differences for processing speed, complex attention, cognitive flexibility and working memory CFQ (ns) Behavioral regulation and metacognition (BRIEF-A) (ns)	Physical and mental fatigue (MFI) (ns)	-	HADS (ns)	-	8/31 5/31
Ho, 2022	-	MMSE (ns) Digit span test (ns) Symbol digit modality test (ns) MoCA (ns)					3/19 3/20
Kozora, 2022	Multitasking, perceptual discrimination tasks, sensorimotor navigation (processing speed and attention)	TMT-A (+),TMT-B (+), WAIS-IV (ns), reaction time (+), Digit vigilance test (ns), Stroop color test (ns)	VAS fatigue and pain (ns)	-	VAS measures of depression and Anxiety (ns)	-	None discontinued
Liu, 2022	-	MoCA (ns), TMT-A (ns), DSST (+), DST (ns), VFT (ns)	-	Barthel index (+)	-	-	None discontinued
Park, 2022	Spatial cognitive training	WAIS-BDT (+) SVLT (+ recall but not recognition)	-	-	-	-	None discontinued
Pottgen, 2022	Working memory and metacognition	Perceived Deficit Questionnaire (ns), working memory (+)	Fatigue Scale for Motor and Cognitive Functions (ns)	FAI (-)	HADS depression and anxiety (ns)	HAQUAMS (ns)	17/68 9/108
Von Ah, 2022	-	PROMIS applied cognition Abilities (ns) Other cognitive measures (ns)	-	work ability (n.s.)	-	SF-36 perceived Change in health (+)	3/22 6/24
Baldimtsi, 2023	20 numerical calculation; memory game	MMSE (+), RAVLT (+), WAIS (+), TMT-B (+)	-	-	-	-	-

Feinstein, 2023	Processing speed	SDMT (ns), CVLT-II (ns), Brief visuospatial memory test (ns)	6MWT (ns), fatigue impact scale (ns), 12-item MS walking scale (ns)	global functional (functional assessment of MS) (ns)	HADS-D/A (ns)	EuroQol-5 Dimensions VAS (ns)	3/79 6/75
Galperin, 2023	Cognitive and gait training	SDMT (+), CVLT-II (ns), WLG (+), TMT-A (+), TMT-B (ns)	MFIS (ns), Dual task gait speed (ns), 25-Foot Walk (T25FW) (ns)	Multiple Sclerosis Walking Scale-12 (MSWS-12) (-)	PHQ-9 (+)	MSQOL-54 mental (+)	12/64 4/60
Goumopoulos, 2023	Semantic, episodic, auditory and visual memory, plus working memory	MoCA (+), RAVLT (+), TMT-A/B (+), DST (+), CDT (ns)	-	Functional activities questionnaire (+) IADL (ns)	GDS (ns)	-	1/11 0/10
Kim, 2023	Attention and executive function, memory, and visuospatial function	MoCA (+), DST (+),CWST (ns)	-	-	GDS (+)	-	-
Maeir, 2023	attention, speed of processing, visual working memory and attentional control	FACTcog (+)	-	COPM (+)	-	FACT-GP (ns)	9/25 10/24
Nousia, 2023	Several cognitive domains, mainly language and memory	MoCA(+), delayed memory(+), digit span (+), BNT(+), Semantic Fluency (+), CDT (+), word recognition (ns), TMT-A/B (ns)	-	-	-	-	4/19 2/17
Baik, 2024	attention, visual perception, memory, and executive functions	MoCA (+), verbal learning tests, DST, semantic fluecy, phonemic fluency,	-	-	GDS (+)	-	None discontinued
Klaver, 2024	Psychoeducation, fatigue management, coping with consequences of cognitive problems, and communication strategies	Cognitive Symptom Checklist-Work Dutch Version (ns)	-	Work Ability Index (ns), Work Role Functioning Questionnaire, VBBA	-	SF-36 general health perception (ns)	21/93 9/93

Supplementary table 3. Outcome measures. Psychometric properties of the most common outcome measures included in the studies.

Scale	Range, direction
Cognition	
Paced Auditory Serial Addition Test (PASAT) (auditory information processing speed and flexibility, as well as calculation ability)	(0-60, higher is better)
Mini Mental Status Examination (MMSE) (global cognition)	(0-30, higher is better)
California Verbal Learning Test - II (CVLT-II) (verbal learning and episodic memory)	(16+16, higher is better)
Repeatable Battery for Assessment of Neuropsychological Status (RBANS) (immediate memory, visuospatial/constructional, language, attention, delayed memory)	(40-160, higher is better)
Symbol digit modality test (attention, perceptual speed, motor speed and visual scanning)	(max = 110, higher is better)
Cambridge Neuropsychological Test Automated Battery (CANTAB) (spatial Working Memory Task)	(higher is better)
Addenbrooke's cognitive examination (ACE) (global cognition)	(0-100, higher is better)
MoCA (global cognition)	(0-30, higher is better)
Barrow Neurological Institute Screen for Higher Cerebral Functions	(0-50, higher is better)
Stroop test (cognitive interference and selective attention)	(0-10, higher is better)
Digit-Span Forward/backward Test (verbal short term and working memory)	(higher is better)
Rey Auditory Verbal Learning Test (RAVLT) (immediate memory)	(0 to 69, higher is better)
Rey-Osterrieth Complex Figure Test (RCFT) (visuospatial function)	(higher is better)
FACT-COG perceived cognitive impairment	(0-148, higher is better)
Spatial recall test (visuospatial memory)	(higher is better)
Memory binding test from Grober and Buschke (Short-term memory)	(higher is better)
Working memory (Block span test) - Corsi block-tapping test	(max 9, higher is better)
Mean reaction time	(lower is better)
Executive Interview 25 (EXIT-25) (executive functions)	(0-50, lower is better)
ASRS-Inattention	(lower is better)
Cognitive Symptom Checklist-Work Dutch Version	(0-100, lower is better)
Perceived Deficit Questionnaire (PDQ)	(lower is better)
Working Memory Questionnaire	(0-120, lower is better)
Performance in activities of daily living	
Timed instrumental ADL	(lower is better)
Functional Activities Questionnaire (FAQ)	(0–30, lower is better)
Bayer-ADL	(lower is better)
Stroke impact scale – ADL	(0 to 100, higher is better)
Modified Barthel Index	(0-100, higher is better)
MSNQ (neuropsychological competence with activities of daily living)	(0-60, lower is better)
Brief Assessment of Prospective Memory IADL subscale	(lower is better)
Work ability index	(0-10, higher is better)
Lawton Instrumental Activities of Daily Living scale (IADL)	(0-8, higher is better)
Sheehan Disability Scale	10-point Likert scale (lower is better)

Canadian Occupational Performance Measure (COPM)	(0-10 for each item, higher is better)
Coping Self Efficacy Scale (CSES)	(higher is better)
Frenchay Activity Index (FAI)	(higher is better)
Functional Assessment of Multiple Sclerosis (FAMS)	(0-176, higher is better)
Multiple Sclerosis Walking Scale-12 (MSWS-12)	(0-100, lower is better)
Work Role Functioning Questionnaire (WRFQ)	(0-100, higher is better)
Experience and assessment of work questionnaire (VBBA)	(0-100, lower is better)
Quality of life	
EuroQol EQ5-VAS	(0–100, higher is better)
Short-Form Survey (SF-12/36)	(0 to 100, higher is better)
Multiple Sclerosis Quality of Life-54 items (MSQOL-54)	(0 to 100, higher is better)
Parkinson's Disease Questionnaire (PDQ-39/PDQ-8)	(0 to 100, lower is better)
Stroke Specific Quality of Life Scale (SS-QoL-12)	(49 to 245, higher is better)
Spitzer QoL	(0-10, higher is better)
FACT-P, Functional Assessment of Cancer Therapy-Prostate	(higher is better)
FACT-Cog QoL	(higher is better)
Functional Assessment of Cancer Therapy-General practice (FACT-GP)	(21 items, higher is better)
Hamburg Quality of Life Questionnaire in Multiple Sclerosis (HAQUAMS)	(lower is better)
Quality of Life-Alzheimer Disease (QoL-AD)	(higher is better)
ADHD Impact Module–Adult (AIM-A)	Higher is better
Depression and anxiety	
Beck depression inventory-II (BDI)	(lower is better)
Hospital Anxiety and Depression Scale (HADS)	(0-21, lower is better)
Geriatric Depression Scale (GDS)	(0-15, lower is better)
Neurobehavioral Symptom Inventory (NSI)	(0 to 88, lower is better)
Montgomery-Asberg Depression Rating Scale (MADRS)	(lower is better)
Center for Epidemiologic Study-Depression (CES-D)	(0-60, lower is better)
State-Trait Anxiety Inventory (STAI)	(20-80, lower is better)
Hamilton Depression Rating Scale (HAMD)	(0-52, lower is better)
Apathy index/evaluation scale (AES)	(18–72, higher is better)
Anxiety/depression - 12-item General Health Questionnaire	(0-84, lower is better)
Positive and Negative Affect Schedule-positive affect (PANAS-P/PANAS-N)	PANAS-P (higher is better)<br>PANAS-N (lower is better)
Patient Health Questionnaire 9 (PHQ-9)	(5-27, lower is better)
Fatigue	
Fatigue Severity Scale (FSS)	(9-63, lower is better)
Fatigue Scale for Motor and Cognitive Functions (FSMC)	(lower is better)
Modified Fatigue Impact Scale (MFIS)	(0-84, lower is better)
FACT Fatigue subscale	(higher is better)

Supplementary table 4. PRISMA 2020 Checklist.

Section and Topic	Item #	Checklist item	Location where item is reported
**TITLE**			
Title	1	Identify the report as a systematic review.	Title
**ABSTRACT**			
Abstract	2	See the PRISMA 2020 for Abstracts checklist.	
**INTRODUCTION**			
Rationale	3	Describe the rationale for the review in the context of existing knowledge.	Pag 4
Objectives	4	Provide an explicit statement of the objective(s) or question(s) the review addresses.	Pag 4
**METHODS**			
Eligibility criteria	5	Specify the inclusion and exclusion criteria for the review and how studies were grouped for the syntheses.	Pag 11-12, table 4
Information sources	6	Specify all databases, registers, websites, organisations, reference lists and other sources searched or consulted to identify studies. Specify the date when each source was last searched or consulted.	Pag 12
Search strategy	7	Present the full search strategies for all databases, registers and websites, including any filters and limits used.	Pag 12 and supplementary table 4
Selection process	8	Specify the methods used to decide whether a study met the inclusion criteria of the review, including how many reviewers screened each record and each report retrieved, whether they worked independently, and if applicable, details of automation tools used in the process.	Pag 11-12
Data collection process	9	Specify the methods used to collect data from reports, including how many reviewers collected data from each report, whether they worked independently, any processes for obtaining or confirming data from study investigators, and if applicable, details of automation tools used in the process.	Pag 12-13
Data items	10a	List and define all outcomes for which data were sought. Specify whether all results that were compatible with each outcome domain in each study were sought (e.g. for all measures, time points, analyses), and if not, the methods used to decide which results to collect.	Pag 12-13, supplementary tables 1, 2, 3
	10b	List and define all other variables for which data were sought (e.g. participant and intervention characteristics, funding sources). Describe any assumptions made about any missing or unclear information.	Pag 12-13, supplementary tables 1, 2, 3
Study risk of bias assessment	11	Specify the methods used to assess risk of bias in the included studies, including details of the tool(s) used, how many reviewers assessed each study and whether they worked independently, and if applicable, details of automation tools used in the process.	Pag 12
Effect measures	12	Specify for each outcome the effect measure(s) (e.g. risk ratio, mean difference) used in the synthesis or presentation of results.	Pag 12-13

Section and Topic	Item #	Checklist item	Location where item is reported
Synthesis methods	13a	Describe the processes used to decide which studies were eligible for each synthesis (e.g. tabulating the study intervention characteristics and comparing against the planned groups for each synthesis (item #5)).	Followed the inclusion and exclusion criteria on Covidence, independently by two authors, pag 11-12
	13b	Describe any methods required to prepare the data for presentation or synthesis, such as handling of missing summary statistics, or data conversions.	Pag 11-12
	13c	Describe any methods used to tabulate or visually display results of individual studies and syntheses.	Pag 12, supplementary data 2 and 3
	13d	Describe any methods used to synthesize results and provide a rationale for the choice(s). If meta-analysis was performed, describe the model(s), method(s) to identify the presence and extent of statistical heterogeneity, and software package(s) used.	Pag 12-13
	13e	Describe any methods used to explore possible causes of heterogeneity among study results (e.g. subgroup analysis, meta-regression).	Pag 12-13
	13f	Describe any sensitivity analyses conducted to assess robustness of the synthesized results.	Pag 13
Reporting bias assessment	14	Describe any methods used to assess risk of bias due to missing results in a synthesis (arising from reporting biases).	Pag 12-13, figure 2, supplementary figure 1
Certainty assessment	15	Describe any methods used to assess certainty (or confidence) in the body of evidence for an outcome.	13
**RESULTS**			
Study selection	16a	Describe the results of the search and selection process, from the number of records identified in the search to the number of studies included in the review, ideally using a flow diagram.	Pag 4-5 and Figure 1
	16b	Cite studies that might appear to meet the inclusion criteria, but which were excluded, and explain why they were excluded.	
Study characteristics	17	Cite each included study and present its characteristics.	Pag 5-6<br>Table 1<br>Supplementary tables 1 and 2
Risk of bias in studies	18	Present assessments of risk of bias for each included study.	Supplementary figure 1
Results of	19	For all outcomes, present, for each study: (a) summary statistics for each group (where appropriate) and (b) an effect estimate and its	Pag 6-8

Section and Topic	Item #	Checklist item	Location where item is reported
individual studies		precision (e.g. confidence/credible interval), ideally using structured tables or plots.	Figures 3-7
Results of syntheses	20a	For each synthesis, briefly summarise the characteristics and risk of bias among contributing studies.	Pag 6-8<br>Figures 3-7
	20b	Present results of all statistical syntheses conducted. If meta-analysis was done, present for each the summary estimate and its precision (e.g. confidence/credible interval) and measures of statistical heterogeneity. If comparing groups, describe the direction of the effect.	Pag 6-8<br>Figures 3-7
	20c	Present results of all investigations of possible causes of heterogeneity among study results.	Pag 6-8<br>Figures 3-7
	20d	Present results of all sensitivity analyses conducted to assess the robustness of the synthesized results.	Pag 7-8
Reporting biases	21	Present assessments of risk of bias due to missing results (arising from reporting biases) for each synthesis assessed.	Pag 6, 8
Certainty of evidence	22	Present assessments of certainty (or confidence) in the body of evidence for each outcome assessed.	Pag 6-8<br>Figures 3-7
**DISCUSSION**			
Discussion	23a	Provide a general interpretation of the results in the context of other evidence.	Pag 8-11
	23b	Discuss any limitations of the evidence included in the review.	Pag 10-11
	23c	Discuss any limitations of the review processes used.	Pag 10-11
	23d	Discuss implications of the results for practice, policy, and future research.	Pag 11
**OTHER INFORMATION**			
Registration and protocol	24a	Provide registration information for the review, including register name and registration number, or state that the review was not registered.	Pag 11
	24b	Indicate where the review protocol can be accessed, or state that a protocol was not prepared.	Pag 11
	24c	Describe and explain any amendments to information provided at registration or in the protocol.	
Support	25	Describe sources of financial or non-financial support for the review, and the role of the funders or sponsors in the review.	Pag 13-14
Competing interests	26	Declare any competing interests of review authors.	Pag 13-14
Availability of data, code and other materials	27	Report which of the following are publicly available and where they can be found: template data collection forms; data extracted from included studies; data used for all analyses; analytic code; any other materials used in the review.	Pag 13

From: Page MJ, McKenzie JE, Bossuyt PM, Boutron I, Hoffmann TC, Mulrow CD, et al. The PRISMA 2020 statement: an updated guideline for reporting systematic reviews. BMJ 2021;372:n71. doi: 10.1136/bmj.n71

Supplementary table 5. Search strategy. Queries used for searching Ovid MEDLINE, Embase and PsycInfo databases.

#	Searches
1	(("cognition*" OR "cognition disorders/complications" OR "executive function*" OR "Memory" OR "Memory" OR "memory, short term*" OR "cognitive dysfunction/prevention and control") OR "memory disorders" OR "cognitive dysfunction" OR "MCI" OR "executive function" OR (("cognit*" AND "ADJ3" AND ("func*" OR "declin*" OR "reduc*" OR "impair*" OR "improve*" OR "deficit*" OR "progress*" OR "perform*")) OR "mental perform*" OR "executive function*" OR "Dementia" OR "alzheimer disease" OR "dement*" OR "alzheimer*"
2	(("cognitive dysfunction" OR ("cognitive" AND "dysfunction") OR "cognitive dysfunction")) AND "rehabilitation*" OR (("cognitive remediation" OR ("cognitive" AND "remediation") OR "cognitive remediation") AND "methods*") OR (("cognitive behavioral therapy" OR ("cognitive" AND "behavioral" AND "therapy") OR "cognitive behavioral therapy") AND "methods*") OR (("cognition" OR "cognition" OR "cognitions" OR "cognitive" OR "cognitively" OR "cognitives") AND "ADJ3" AND "train*") OR "cognitive exercis*" OR "brain train*" OR "memory rehab*" OR "memory enhance*" OR "brain exercis*" OR "cognitive rehab*" OR (("mental" OR "mentalities" OR "mentality" OR "mentalization" OR "mentalization" OR "mentalizing" OR "mentalize" OR "mentalized" OR "mentally") AND "ADJ3" AND "activit*") OR "cognitive intervention*" OR "cognitive motor intervention*" OR "cognition based intervention*" OR "cognitive enrich*" OR "cognitive behavioral therapy" OR "cognitive behavioral therapy" OR ("behavior*" AND "adj1" AND "therap*") OR "cognitive therapy" OR "attentional restoration" OR "cognitive restoration" OR "cognitive remediation" OR "cognitive training" OR "cognitive recovery"
3	"therapy, computer assisted" OR (("therapy, computer assisted" OR ("therapy" AND "Computer-assisted") OR "computer-assisted therapy" OR ("therapy" AND "computer" AND "assisted") OR "therapy computer assisted") AND "methods*") OR (("self-care" OR ("self" AND "care") OR "self-care") AND "methods*") OR "telephone*" OR (("video games" OR ("video" AND "games") OR "video games") AND "psychology*") OR "virtual reality*" OR "telemedicine" OR "telemedicine" OR (("electronical" OR "electronically" OR "electronics" OR "electronics" OR "electronic") AND "referral and consultation") OR "Remote Consultation" OR ("internet based intervention*" OR "Internet-Delivered intervention" OR ("telemedicine" OR "telemedicine" OR "telemedicine s") OR ("australas plant pathol"[Journal] OR "app") OR "computer assist*" OR "digit*" OR "computari*" OR "Health technology" OR ("mhealth s" OR "telemedicine" OR "telemedicine" OR "mhealth") OR "Mobile Health" OR ("telehealth s" OR "telemedicine" OR "telemedicine" OR "telehealth") OR ("telemedicine" OR "telemedicine" OR "ehealth") OR "Digital assistant" OR "Self-guided" OR "self-management" OR "self-care" OR "Remote Consultation" OR "Self-help" OR "self administ*" OR "self direct*" OR "minimal contact" OR "minimal guidance" OR "unguid*" OR "no guidance" OR "Computer-assisted" OR "self instruct*" OR "self manag*" OR "self administ*" OR "self help*" OR "self guid*" OR "self rehab*" OR "self monitor*" OR "Online treatment" OR "conversational agent")
4	"Feasibility Studies" OR "Treatment Outcome" OR "randomized controlled trial" OR "controlled clinical trial" OR ("random allocation" OR ("random" AND "allocation") OR "random allocation" OR "random" OR "randomization" OR "randomized" OR "randomisation" OR "randomisations" OR "randomise" OR "randomised" OR "randomising" OR "randomizations" OR "randomize" OR "randomizes" OR "randomizing" OR "randomness" OR "randoms") OR ("random allocation" OR ("random" AND "allocation") OR "random allocation" OR "random" OR "randomization" OR "randomized" OR "randomisation" OR "randomisations" OR "randomise" OR "randomised" OR "randomising" OR "randomizations" OR "randomize" OR "randomizes" OR "randomizing" OR "randomness" OR "randoms") OR ("placeboes" OR "placebos" OR "placebos" OR "placebo") OR "randomly" OR ("clinical trials as topic" OR ("clinical" AND "trials" AND "topic") OR "clinical trials as topic" OR "trial" OR "trial s" OR "trialed" OR "trialing" OR "trials") OR ("group s" OR "grouped" OR "grouping" OR "groupings" OR "groups" OR "groups s" OR "population groups" OR ("population" AND "groups") OR "population groups" OR "group")
5	1 and 2 and 3 and 4

Supplementary figure 1. Risk of bias summary. Summary of review authors' judgements about each risk of bias item for each included study. Two authors used the revised Cochrane risk-of-bias 2.0 tool for randomized trials containing the following domains: random sequence generation, allocation sequence concealment, blinding of participants and personnel, blinding of outcome assessors, incomplete outcome data, and selective reporting. Based on predefined definition, studies with high risk of bias or some concerns in the different domains were considered as having a high risk of bias. Studies which were rated as low on all available criteria were rated as overall low risk of bias. The inter-rater reliability for the risk of bias assessments showed a Cohen's kappa of 0.70 indicating good agreement.

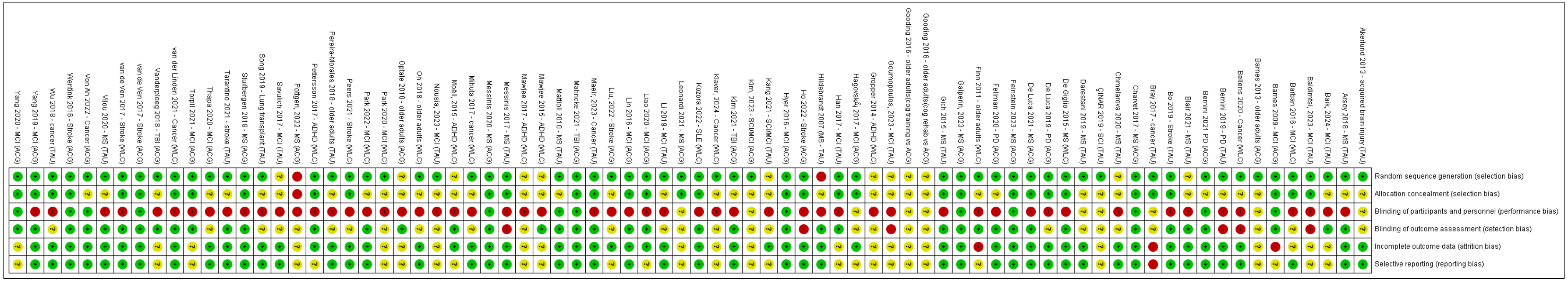

Supplementary figure 2. Subgroup analysis funnel plots. Funnel plot of subgroup analysis of all interventions per disease (a) and delivery format (b) for outcome cognition; a) studies focusing on ADHD (k=5), Parkinson's disease (k=4), MCI/subjective cognitive symptoms (k=28) and multiple sclerosis (k=16), ADHD (k=5), and exhibited significantly greater treatment effects (moderate to large effects), while no benefit was found post-stroke (k=7), in cancer survivors (k=7), post-traumatic brain injury (k=3) and post-lung transplant (k=1), and the difference between groups was statistically significant (p<0.00001); b) trials using virtual reality (k=13) and videogames (k=3) digital interventions appear to exhibit greater treatment benefits for cognitive symptoms in comparison to the other formats, but the difference between groups regarding mode of delivery was not statistically significant (p=0.56).

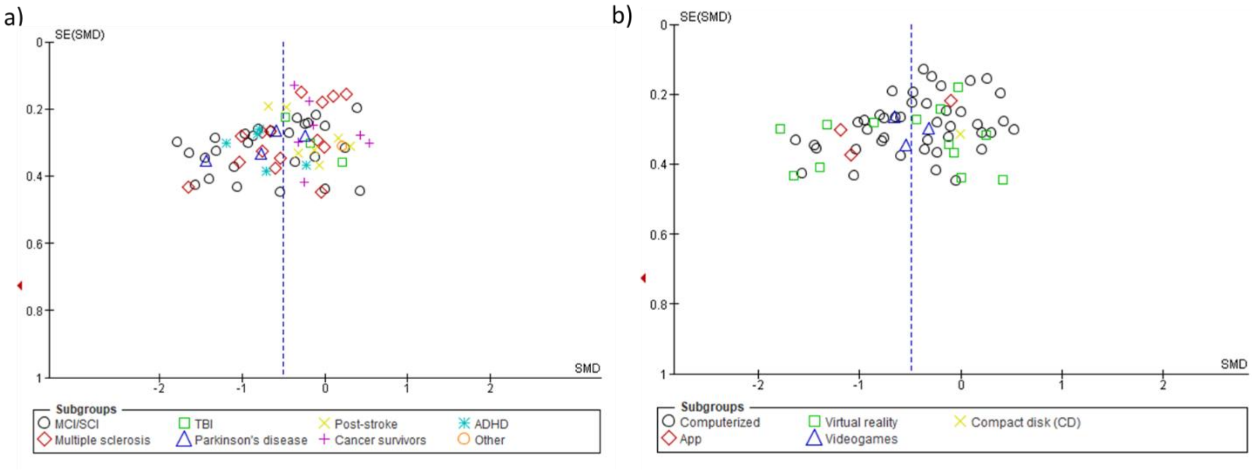

Supplementary figure 3. Funnel plots for cognition. Plots for publication bias assessment of cognitive outcomes (a – cognitive training; b-cognitive rehabilitation; c-virtual reality; d-videogames; e-internet CBT-based programs). The Egger's test was significant for cognitive training (p< 0.0001) and cognitive rehabilitation interventions (p=0.003) and internet CBT-based programs (p < 0.0001).

a)

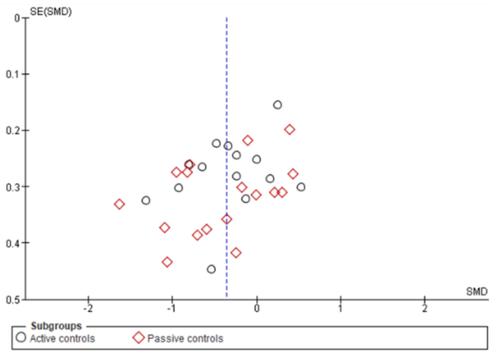

b)

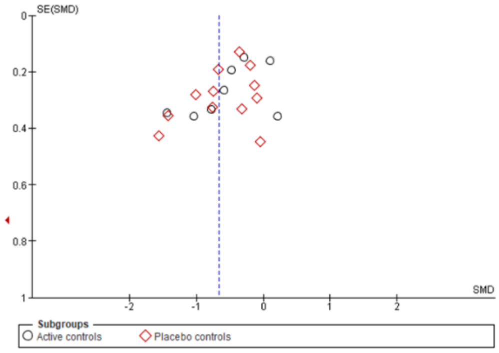

c)

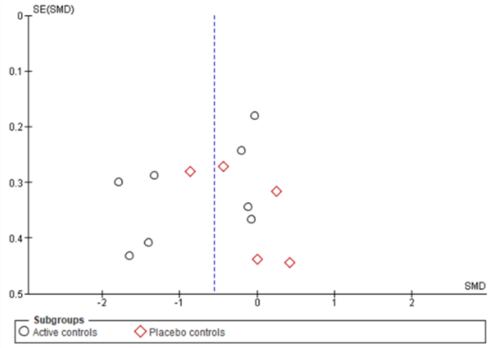

d)

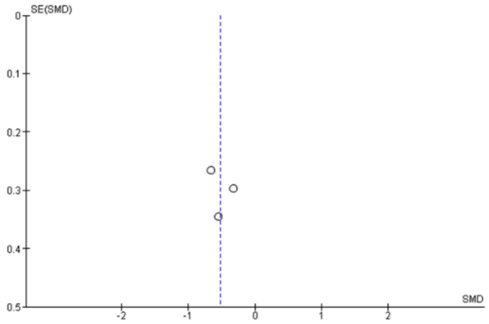

e)

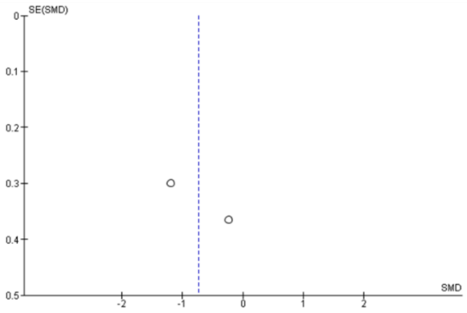

Supplementary figure 4. Funnel plots for physical health/fatigue outcome. Plots for publication bias assessment of fatigue/physical health outcomes (a – cognitive training; b- cognitive rehabilitation). No significant publication bias was found.

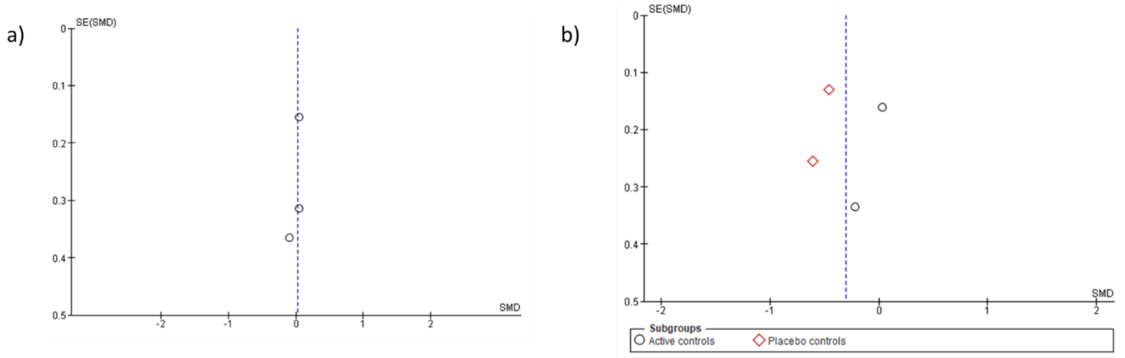

Supplementary figure 5. Funnel plots for performance in activities of daily living. Plots for publication bias assessment of performance in activities of daily living outcomes (a – cognitive training; b-cognitive rehabilitation; c-virtual reality; d-internet CBT-based programs). No significant publication bias was found.

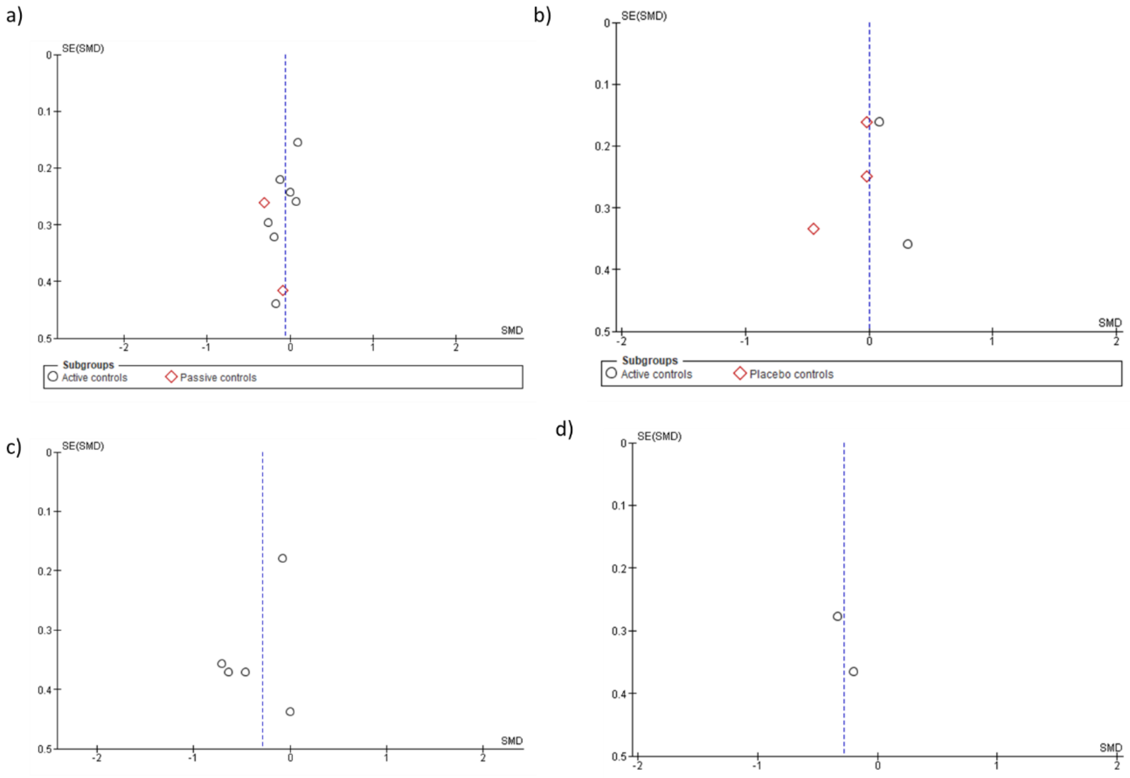

Supplementary figure 6. Funnel plots for mental health. Plots for publication bias assessment of mental health outcomes (a – cognitive training; b-cognitive rehabilitation; c-virtual reality; d-internet CBT-based programs). The Egger's test suggests that unpublished studies with opposing effects may eventually exist for cognitive training (p=0.04).

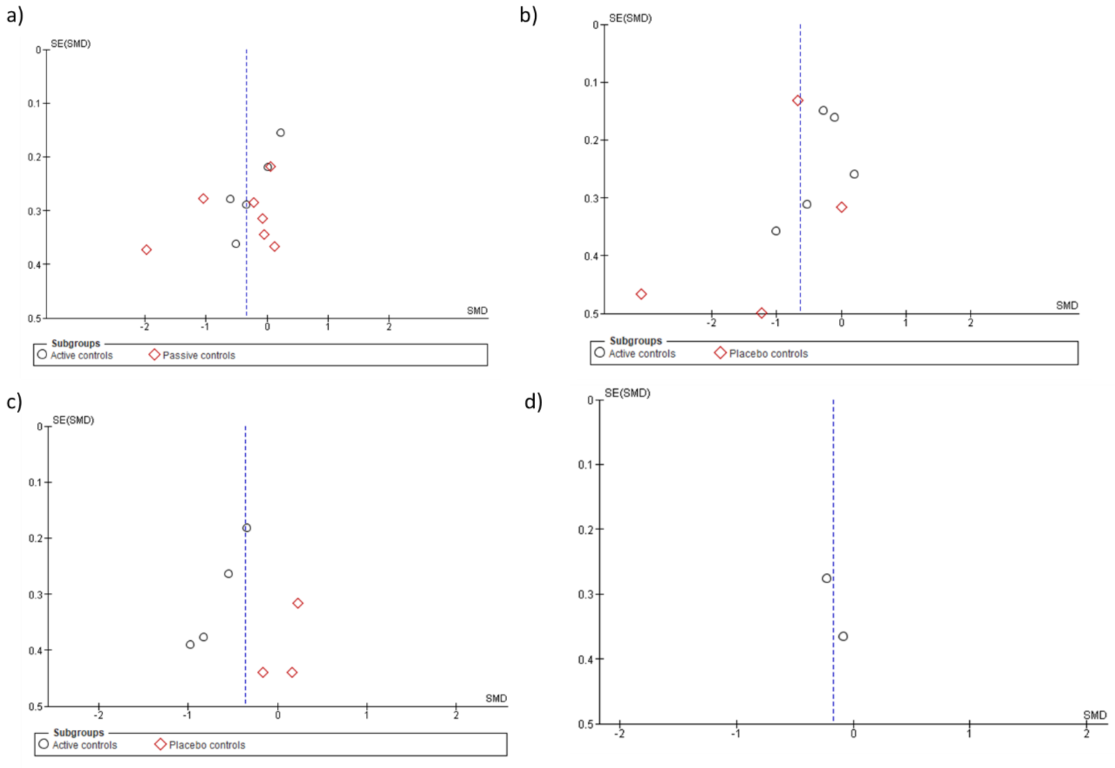

Supplementary figure 7. Funnel plots for quality of life. Plots for publication bias assessment of quality of life outcomes (a – cognitive training; b-cognitive rehabilitation; c-virtual reality; d-videogames). No significant publication bias was found.

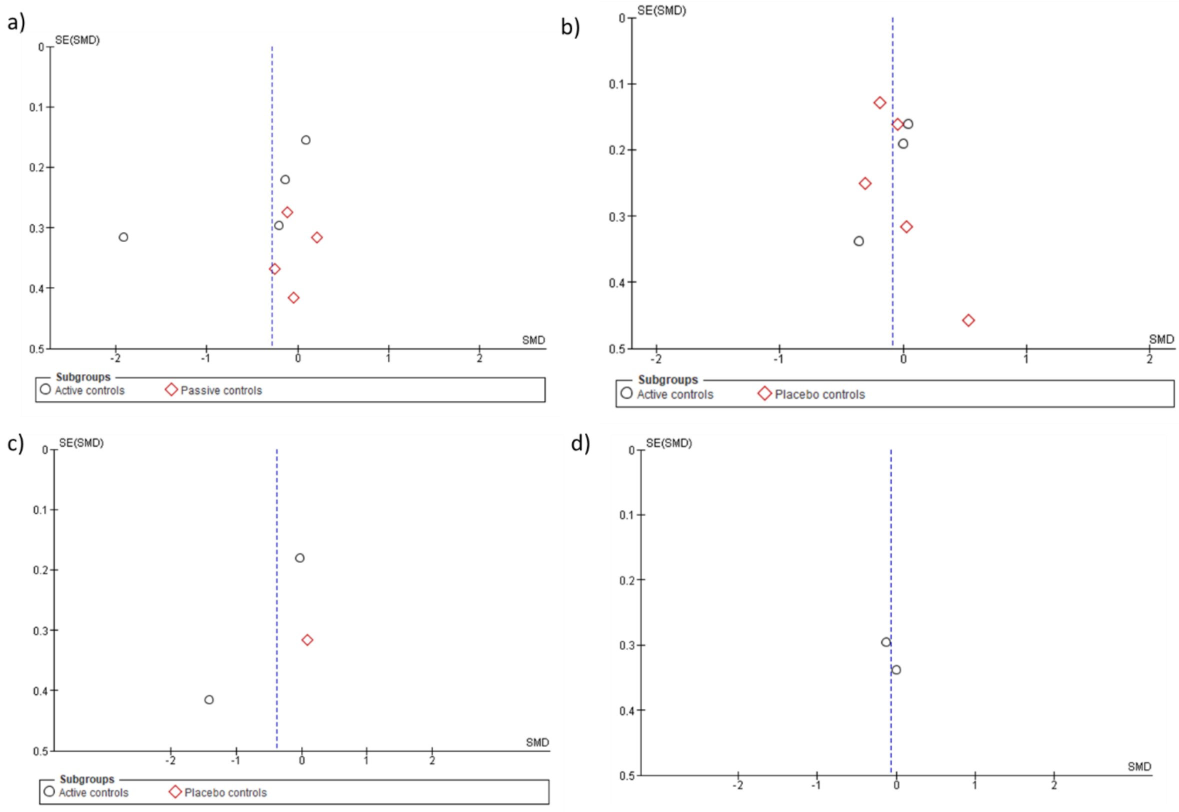

Supplementary data 1. Extracted data for each individual study. Spreadsheet file containing detailed information about each individual study.

Supplementary data 2. Metadata for metanalysis. Spreadsheet file containing data for metanalysis calculation, containing data per individual treatment framework and outcome.
